# Genome Reduction in *Psychromonas* Species within the Gut of an Amphipod from the Ocean’s Deepest Point

**DOI:** 10.1128/mSystems.00009-18

**Published:** 2018-04-10

**Authors:** Weipeng Zhang, Ren-Mao Tian, Jin Sun, Salim Bougouffa, Wei Ding, Lin Cai, Yi Lan, Haoya Tong, Yongxin Li, Alan J. Jamieson, Vladimir B. Bajic, Jeffrey C. Drazen, Douglas Bartlett, Pei-Yuan Qian

**Affiliations:** a Division of Life Science, Hong Kong University of Science and Technology, Kowloon, Hong Kong, China; b Computational Bioscience Research Center, King Abdullah University of Science and Technology, Thuwal, Saudi Arabia; c School of Marine Science and Technology, Newcastle University, Newcastle Upon Tyne, Tyne and Wear, United Kingdom; d Department of Oceanography, University of Hawaii at Manoa, Honolulu, Hawaii, USA; e Marine Biology Research Division, Scripps Institution of Oceanography, University of California San Diego, La Jolla, California, USA; Dalhousie University

**Keywords:** amphipod, gut microbiota, Mariana Trench, *Psychromonas*

## Abstract

Amphipods are the dominant scavenging metazoan species in the Mariana Trench, the deepest known point in Earth’s oceans. Here the gut microbiota of the amphipod Hirondellea gigas collected from the Challenger and Sirena Deeps of the Mariana Trench were investigated. The 11 amphipod individuals included for analyses were dominated by *Psychromonas*, of which a nearly complete genome was successfully recovered (designated CDP1). Compared with previously reported free-living *Psychromonas* strains, CDP1 has a highly reduced genome. Genome alignment showed deletion of the trimethylamine *N*-oxide (TMAO) reducing gene cluster in CDP1, suggesting that the “piezolyte” function of TMAO is more important than its function in respiration, which may lead to TMAO accumulation. In terms of nutrient utilization, the bacterium retains its central carbohydrate metabolism but lacks most of the extended carbohydrate utilization pathways, suggesting the confinement of *Psychromonas* to the host gut and sequestration from more variable environmental conditions. Moreover, CDP1 contains a complete formate hydrogenlyase complex, which might be involved in energy production. The genomic analyses imply that CDP1 may have developed adaptive strategies for a lifestyle within the gut of the hadal amphipod H. gigas.

**IMPORTANCE** As a unique but poorly investigated habitat within marine ecosystems, hadal trenches have received interest in recent years. This study explores the gut microbial composition and function in hadal amphipods, which are among the dominant carrion feeders in hadal habitats. Further analyses of a dominant strain revealed genomic features that may contribute to its adaptation to the amphipod gut environment. Our findings provide new insights into animal-associated bacteria in the hadal biosphere.

## Abstract (precis)

As a unique but poorly investigated habitat within marine ecosystems, hadal trenches have received interest in recent years. This study explores the gut microbial composition and function in hadal amphipods, which are among the dominant carrion feeders in hadal habitats. Further analyses of a dominant strain revealed genomic features that may contribute to its adaptation to the amphipod gut environment. Our findings provide new insights into animal-associated bacteria in the hadal biosphere.

## INTRODUCTION

The hadal zone is the deepest area of the ocean (6,000 to 11,000 m), which exists in long but narrow topographic V-shaped depressions and constitutes the deepest 45% of the vertical depth gradient (1–3). The hadal zone is characterized by low temperatures, seafloor topography, and in particular, high pressures. Few studies have investigated microbial communities in hadal waters (4, 5) and sediments (6). These studies have revealed the involvement of microbial communities in carbon turnover, sulfur cycling, and methane and hydrogen utilization. On the basis of 16S rRNA gene sequencing, it has been known that within the Challenger Deep of the Mariana Trench, the deepest point of the hadal zone, bacterial communities were dominated by a wide diversity of *Gammaproteobacteria*, while differences between particle-associated and free-living communities were reflected by variations of *Betaproteobacteria*, *Deltaproteobacteria*, *Chlorobi*, *Atribacteria*, *Chloroflexi*, *Marinimicrobia*, *Cyanobacteria*, *Gemmatimonadetes*, and *Nitrospirae* (5). However, the lack of metagenome and genome information hinders the understanding of microbial adaptation in hadal environments. In particular, genomic analysis of specific groups of animal gut microbiota within such deep environments is rarely performed.

The animal gut microbiota has been shown to be involved in a number of aspects of normal host physiology, such as nutritional status and environmental adaptation (7–11), and consequently the environment of the host can affect the gut microbial composition. Specifically, in human beings, changes in gut microbial composition modulate plasma tryptophan concentrations, which affect neurotransmission within both the enteric and central nervous systems (10). In a study on two euryhaline species, Oreochromis niloticus (Nile tilapia) and Litopenaeus vannamei (Pacific white shrimp), the change in salinity is linked to the shift in the gut microbial composition, possibly because of the salinity stress response of the host and subsequent stress exerted on the associated microbes (11).

On the basis of records from extensive deep-sea sampling, amphipods (Arthropoda: Crustacea: Amphipoda) are among the dominant carrion feeders in hadal habitats. Scavenging amphipods have been studied from the deep sea at depths ranging from 4,855 to 10,897 m in the northeast Atlantic Ocean (12), the central Pacific Ocean (13), the North Pacific Gyre (14), the Izu-Bonin Trench (15), the Kermadec Trench (16), the Peru-Chile Trench (17), the New Hebrides Trench (18), the Philippine Trench (19), and the Mariana Trench (20). The wide distribution of amphipods reflects their success in the deep sea, and thus the unique environmental factors may have contributed to the evolution and existence of specific microbial species in the guts of deep-sea amphipods.

One of the dominant amphipod species present at depths of >10,000 m is Hirondellea gigas (19), which possesses a number of microorganisms in its gut. In the present study, we analyzed the gut metagenomes of 11 H. gigas individuals from the Mariana Trench, including 5 from the Challenger Deep (CD1 to CD5) and six from the Sirena Deep (SD1 to SD6). We further reconstructed a nearly complete genome of the dominant bacterium, a strain belonging to the genus *Psychromonas*, members of which are psychrophilic bacteria that inhabit a number of marine environments (21–23) but have never been found at a >10,000-m depth. Comparative genomics of the predominant *Psychromonas* population (strain CDP1) showed significant genomic reduction and host-specific adaptive traits, possibly indicating a long evolutionary history between these organisms.

## RESULTS

### Taxonomy and function of the gut communities.

**FIG 1:**
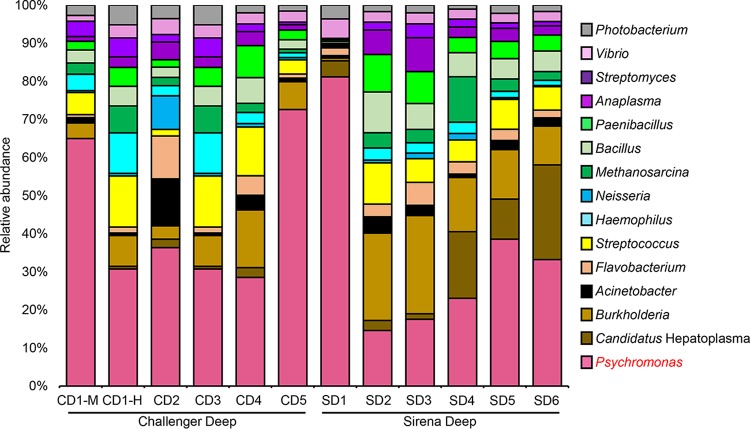
Microbial taxonomic structures deduced from the gut metagenomes. The community compositions are displayed at the genus level based on MEGAN classification. *Psychromonas* is the dominant genus in most of the samples from the Challenger Deep (CD) and the Sirena Deep (SD). One of the Challenger Deep gut samples was separated into midgut (CD1-M) and hindgut (CD1-H) samples. Genera totaling >1% of the samples are shown.

**FIG 2:**
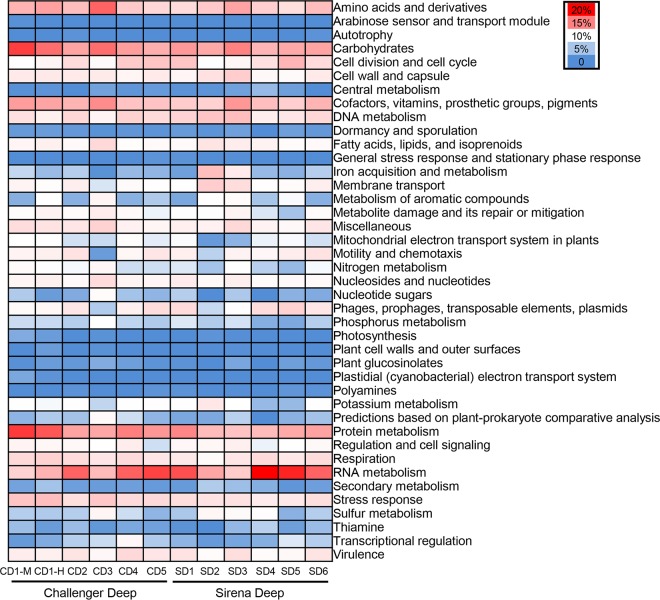
Microbial taxonomic structures deduced from the gut metagenomes. Functional categories are displayed at the SEED1 (the most general categories) level.

The species affiliation of H. gigas was confirmed through phylogenetic analysis of the cytochrome *c* oxidase subunit I gene from the mitochondrial genome (24). For one of the H. gigas individuals from the Challenger Deep (CD1), the gut was divided into mid and hind parts, samples of which were sequenced separately (this was done to facilitate genome binning from two related metagenomes), while the midgut and hindgut samples of the other H. gigas strains were sequenced together. In total, 12 metagenomes with paired-end reads (150 bp in length) were obtained, amounting to 163.2 Gb of data. Read numbers, trimming and quality filtering, and assembled contigs are summarized in Table S1 in the supplemental material. The community composition deduced from the metagenomes revealed that bacterial reads accounted for 11.7 to 16.2% of the total reads, whereas archaea accounted for <0.1%. The rest of the DNA reads were from the amphipod DNA. At the genus level, a total of 15 taxa with >1% relative abundance of the total bacteria and archaea were identified in all of the metagenomes, such as *Psychromonas*, “*Candidatus* Hepatoplasma,” and *Burkholderia* (Fig. 1). SEED-based functional classification of the microbial genes revealed the prevalence of genes for the metabolism of a number of carbon sources, such as carbohydrate, protein, DNA, and RNA (Fig. 2). Moreover, genes for anaerobic respiration accounted for >10% of the metagenomes. Almost no genes for autotrophy and photosynthesis were detected. Overall, similar functional patterns were observed for all of the amphipod individuals analyzed. In addition, clustered regularly interspaced short palindromic repeats (CRISPRs) were identified in microbial contigs of four metagenomes (CD2, CD3, SD2, and SD3) (Table S2), suggesting that viral pressure might be present within some of the amphipod hosts.

10.1128/mSystems.00009-18.5 TABLE S1 The sequence profile and quality control of the metagenomic and metatranscriptomic paired-end reads of the amphipod gut samples (CD, Challenger Deep; SD, Sirena Deep) sequenced with the Illumina HiSeq 2500 platform. Download TABLE S1, DOCX file, 0.02 MB. Copyright © 2018 Zhang et al. 2018 Zhang et al. This content is distributed under the terms of the Creative Commons Attribution 4.0 International license.

10.1128/mSystems.00009-18.6 TABLE S2 CRISPRs identified in the microbial contigs of the gut metagenomes. Download TABLE S2, DOCX file, 0.02 MB. Copyright © 2018 Zhang et al. 2018 Zhang et al. This content is distributed under the terms of the Creative Commons Attribution 4.0 International license.

### Microscopic images of the amphipod gut microbiota.

Transects of the gut of one H. gigas specimen from the Challenger Deep were approximately 0.5 mm in diameter (Fig. S1A). The microbial cells were generally rod shaped or cocci (1 to 2 µm) attached to the surface of the gray-stained feces. Fluorescence *in situ* hybridization (FISH) demonstrated that most of the microbes in the gut were bacteria rather than archaea (Fig. S1B), consistent with the sequencing results. *Psychromonas* cells were stained by the Cy3-labeled specific probe and appeared blue in the FISH experiment (Fig. S1C). The negative control using an unrelated probe showed no signals (Fig. S1D). These images of the gut microbiota confirmed the presence of gut microbes, as well as the dominance of the *Psychromonas* strain (designated CDP1).

10.1128/mSystems.00009-18.1 FIG S1  Morphological analysis of the gut microbiota. (A) A whole transect profile of the gut stained by DAPI, displaying the gut wall and content. (B) FISH image showing specific probe staining for all bacteria. (C) FISH image obtained with the *Psychromonas*-specific probe. (D) Negative-control image obtained with an unrelated probe. Download FIG S1, DOCX file, 2.4 MB. Copyright © 2018 Zhang et al. 2018 Zhang et al. This content is distributed under the terms of the Creative Commons Attribution 4.0 International license.

### General features of the *Psychromonas* genome from the Challenger Deep.

*Psychromonas* was the most dominant genus in the gut microbiota, and it accounted for 12.5 to 60.5% of the total community (Fig. 1). Conserved single-copy gene comparison suggested that there was only one *Psychromonas* strain in each gut; strains from different amphipod individuals harbored 16S rRNA genes with nearly identical (>99% similarity) sequences, and their close evolutionary relationship was confirmed by phylogenetic analysis (Fig. S2). Consistent with the 16S rRNA gene result, genomic average nucleotide identities (ANIs) of >97% were detected between CDP1 and the 10 *Psychromonas* genomes from the other 10 amphipod gut metagenomes (Table S3). After genome binning, reassembly, and gap closing, the nearly complete genome contained three contigs with a total length of 1,688,241 bp. The completeness and contamination of the draft genome were estimated as 99.3 and 0%, respectively, on the basis of calculation of the 139 conserved single-copy genes (Table S4).

10.1128/mSystems.00009-18.2 FIG S2  Maximum-likelihood tree based on partial 16S rRNA genes (alignment length of 1,545 bp) recovered from metagenomes showing the phylogenetic relationship of *Psychromonas* strains from different amphipod guts. The 16S rRNA gene of P. arctica DSM 14288 was used to root the tree, and a bootstrap replication number of 500 was used for calculation. Download FIG S2, DOCX file, 0.02 MB. Copyright © 2018 Zhang et al. 2018 Zhang et al. This content is distributed under the terms of the Creative Commons Attribution 4.0 International license.

10.1128/mSystems.00009-18.7 TABLE S3 Information about the *Psychromonas* draft genomes extracted from the 10 metagenomes (except CD1-M and CD1-H). The ANIs between these genomes and CDP1 are shown. Download TABLE S3, DOCX file, 0.01 MB. Copyright © 2018 Zhang et al. 2018 Zhang et al. This content is distributed under the terms of the Creative Commons Attribution 4.0 International license.

10.1128/mSystems.00009-18.8 TABLE S4 Distribution of the 139 hidden Markov models of single-copy protein-coding genes in the CDP1 and reference bacterial genomes. Download TABLE S4, DOCX file, 0.02 MB. Copyright © 2018 Zhang et al. 2018 Zhang et al. This content is distributed under the terms of the Creative Commons Attribution 4.0 International license.

### Phylogenetic location, central metabolic pathway, and gene expression.

**FIG 3:**
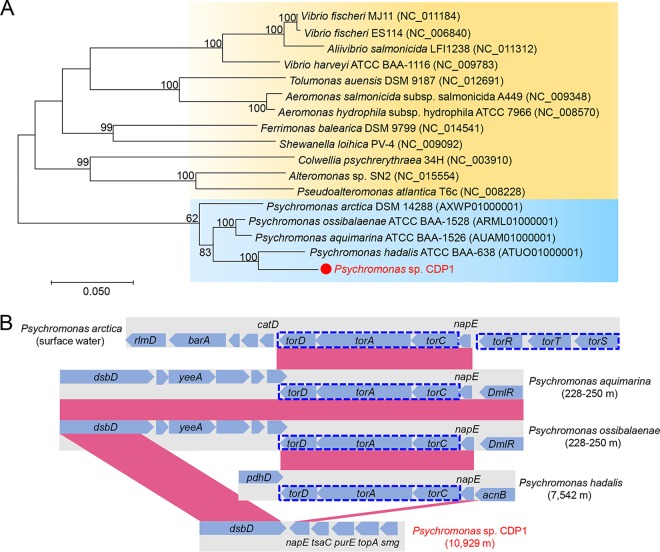
Phylogenetic location of CDP1 and deletion of the *torCAD* operon. (A) Maximum-likelihood phylogenetic tree of 27 concatenated marker genes from CDP1 and the reference genomes. The *Psychromonas* strains can be divided into two branches, the deep-sea lineage (>5,000-m depth) and the shallow-water lineage (0- to 250-m depth). CDP1 is indicated by a solid red circle and located in the deep-sea lineage. A bootstrap replication number of 500 was used in the calculation. (B) Deletion of the *torCAD* operon (boxed in blue broken lines) in CDP1 compared with other *Psychromonas* strains. The *tor* genes could not be found elsewhere in the genomes or metagenomes.

We next compared the CDP1 genome with closely related bacterial genomes. Phylogenetic analysis with 27 conserved single-copy genes revealed two branches for *Psychromonas*, one from the deep sea (>5,000-m depth) and the other from shallow water (0- to 250-m depth) (Fig. 3A). CDP1 was localized with Psychromonas hadalis isolated from seawater at a depth of 7,500 m (21). The other branch included Psychromonas ossibalaenae (22), Psychromonas aquimarina, and Psychromonas arctica (23), which were isolated from seawater at a depth 0 to 250 m. Four representative free-living bacteria from the same order (*Alteromonadales*) were also analyzed as references, and general features of the above-mentioned genomes are summarized in Table 1. The central metabolism of CDP1 included genes involved in carbohydrate metabolism, including complete glycolytic and citric acid cycle pathways, which suggested heterotrophic metabolism. The information of the transcriptomes is summarized in Table S1. Expression of carbohydrate metabolism genes was evident in CDP1 from both the midgut and the hindgut, as evaluated by read mapping and CAZy classification (Fig. S3A and B). These results suggested that CDP1 is indeed active within the gut environment. The 30 clusters of orthologous groups (COGs) with the highest expression level were also listed (Fig. S3C and D), including several genes involved in DNA replication (e.g., COG0305) and ATP synthesis (e.g., COG0056). Overexpression of genes with such functions was also revealed in a previous study investigating the deep-sea microbial biosphere (25), suggesting that the metatranscriptomes in the present study can most likely reflect *in situ* gene expression status.

10.1128/mSystems.00009-18.3 FIG S3  The top 20 active CAZy (A, B) and top 30 COG (C, D) categories in *Psychromonas* sp. strain CDP1 based on mapping of the midgut and hindgut metatranscriptomic reads to the CDP1 genome. RPKM, reads per kilobase per million mapped reads. Download FIG S3, DOCX file, 0.1 MB. Copyright © 2018 Zhang et al. 2018 Zhang et al. This content is distributed under the terms of the Creative Commons Attribution 4.0 International license.

**TABLE 1:** Comparison of general features of the CDP1 and reference genomes^a^

Parameter	*Psychromonas* sp. strain CDP1	*P. hadalis*	*P. ossibalaenae*	*P. aquimarina*	*P. arctica*	*Alteromonas macleodii*	*Colwellia piezophila*	*Pseudoalteromonas* sp.	*Shewanella violacea*
Genetic lineage	I	I	II	II	II				
Habitat	Gut	Free living	Free living	Free living	Free living	Free living	Free living	Free living	Free living
Depth (m)	10,929	7,542	228–250	228–250	0	3,500	6,278	1,855	5,110
NCBI accession no.	NDFH00000000	ATUO01000001	ARML01000001	AUAM01000001	AXWP01000001	CP004851	ARKQ01000000	CP001796	NC_014012
Genome completeness (%)	99.3	100.0	100.0	100.0	100.0	100.0	100.0	100.0	100.0
Genome size (Mbp)	1.69	3.98	5.20	5.53	4.73	4.44	5.48	3.33	4.96
GC content (%)	36.9	39.1	42.0	42.5	37.8	44.7	52.1	40.2	44.7
No. of coding sequences	1,588	3,724	4,480	4,772	4,081	3,898	4,511	3,604	4,081
Avg intergenic length (bp)	95.5	138.9	228.1	197.2	167.6	135.3	193.2	119.8	196.6

a The *Psychromonas* references include P. hadalis ATCC BAA-638, P. ossibalaenae ATCC BAA-1528, P. aquimarina ATCC BAA-1526, and P. arctica DSM 14288. The four genomes from the same order used for comparison were those of Alteromonas macleodii U7, Colwellia piezophila BAA-637, *Pseudoalteromonas* sp. strain SM9913, and Shewanella violacea DSS12.

### Smaller genome because of fewer coding sequences and shortened intergenic regions.

CDP1 contained a greatly reduced genome totaling less than 50% of those of other closely related planktonic *Psychromonas* populations. Specifically, CDP1 had 1,588 coding sequences; the other four *Psychromonas* genomes had an average of 3,911 coding sequences, whereas the four non-*Psychromonas* genomes from the order *Alteromonadales* had an average of 4,024 coding sequences (Table 1). Another unique feature of CDP1 was intergenic length reduction. The intergenic regions of the CDP1 genome had been reduced to a large degree, exhibiting an average length of only 95.5 bp; the average intergenic length in the other four *Psychromonas* genomes was 183.0 bp, whereas that of the four non-*Psychromonas* references was 161.2 bp (Table 1).

### Deletion of the TMAO-reducing genes.

To further illuminate genome reduction in CDP1, we investigated its unique genomic features and attempted to link these features with environmental adaptation. The structural comparison of CDP1 and the reference genomes revealed a number of gene deletions in the CDP1 genome. Through manual inspection, we observed that the *torCAD* operon was missing from CDP1, as indicated by the alignment of the flanking regions of this operon (Fig. 3B). This operon in P. hadalis, P. ossibalaenae, and P. aquimarina contained *torC*, *torA*, and *torD* sequentially. These three genes encode the *c*-type cytochrome TorC, the periplasmic molybdoenzyme TorA, and the TorA-specific chaperone TorD, respectively, which reduce trimethylamine *N*-oxide (TMAO) to volatile trimethylamine (TMA) (26, 27). The genome of P. arctica, which was from marine surface water, contains the most complete TMAO-reducing gene cluster composed of *torD*, *torA*, *torC*, *torR*, *torT*, and *torS*. Also, *tor* genes could not be found elsewhere in the CDP1 genome or the H. gigas gut metagenomes. Figure 3 reveals the potential influence of water depth and phylogenetic distance on deletion of the *tor* operon, but further demonstration of this notion requires a larger-scale investigation of *Psychromonas* genomes.

### Reduction of genes for carbohydrate metabolism.

**FIG 4:**
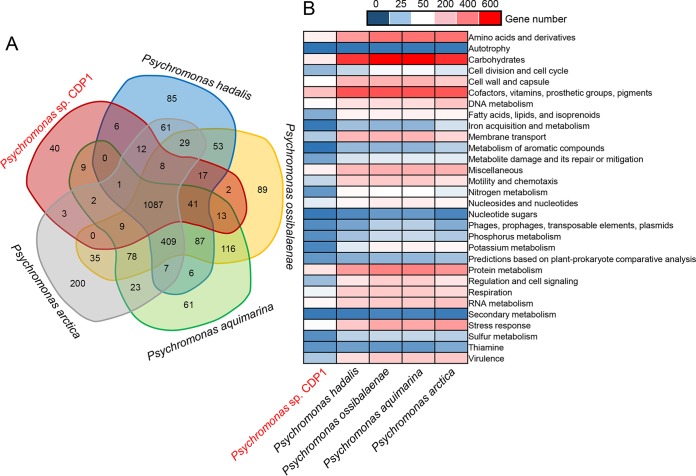
Functional comparison of CDP1 and the reference *Psychromonas* strains. (A) Venn diagram showing the distribution of KEGG genes in the four representative reference genomes. (B) Heat map showing the numbers of genes in different functional categories in CDP1 and the four reference genomes of free-living *Psychromonas* strains. The functional categories were determined by SEED classification after a BLASTP search of the NCBI NR database.

A total of 1,091 KEGG genes were common to the five *Psychromonas* genomes (Fig. 4A). The four reference species (P. hadalis, P. ossibalaenae, P. aquimarina, and P. arctica) had 85, 89, 61, and 200 unique KEGG genes, respectively; however, CDP1 from the Challenger Deep harbored only 40 unique KEGG genes. In addition to genes of the formate hydrogenlyase complex, which will be mentioned again later, these unique KEGG genes in CDP1 included genes for element transport, such as the nitrite transporter gene *nirC*, the ferrous iron transporter gene *feoC*, the potassium uptake protein gene *kup*, and the arsenite-transporting ATPase gene *arsA* (Table S5). These genes may contribute to the host gut-microbiota metabolic interactions through exchange of nutrient elements.

10.1128/mSystems.00009-18.9 TABLE S5 The 40 unique KEGG genes in the CDP1 genome compared with the four reference genomes. Download TABLE S5, DOCX file, 0.01 MB. Copyright © 2018 Zhang et al. 2018 Zhang et al. This content is distributed under the terms of the Creative Commons Attribution 4.0 International license.

SEED classification revealed a reduction of the number of carbohydrate metabolism genes in CDP1 (Fig. 4B). The genome of CDP1 possessed 185 genes for carbohydrate metabolism, whereas the other four *Psychromonas* genomes had >500 genes for carbohydrate metabolism, and the statistical significance of these differences was confirmed by a Mann-Whitney U test (*P* < 0.05). Notably, this reduction in carbohydrate metabolism genes in CDP1 was biased, with central carbohydrate metabolism (glycolysis, citric acid cycle, and acetyl coenzyme A metabolism) being mostly retained and extended carbohydrate metabolism (e.g., alpha-amylase, deoxyribose and deoxynucleoside catabolism, d-gluconate and ketogluconate metabolism, mixed acid fermentation, lactose and galactose uptake and utilization, and trehalose biosynthesis) being greatly reduced (Table S6). Moreover, Fig. 4B shows a reduction of nitrogen metabolism-related genes in CDP1. Further comparison indicated that genes for ammonia, nitrate, and nitrite transport were absent from CDP1 but present in all four of the reference genomes (Table S6). For example, all four of the reference genomes possessed two ammonia transporters, which are used by prokaryotes to scavenge NH_4_^+^/NH_3_, a preferred nitrogen source for cell growth (28); the absence of ammonia transporters from CDP1 may suggest nitrogen source uptake in a different way.

10.1128/mSystems.00009-18.10 TABLE S6 Comparison of functional genes for carbohydrate and nitrogen metabolism in the CDP1 and reference genomes at SEED subsystem level 3. Download TABLE S6, DOCX file, 0.02 MB. Copyright © 2018 Zhang et al. 2018 Zhang et al. This content is distributed under the terms of the Creative Commons Attribution 4.0 International license.

### Complete formate hydrogenlyase complex.

CDP1 and all of the reference *Psychromonas* strains contained formate dehydrogenase, but only CDP1 contained a formate hydrogenlyase complex (Table 2). The formate hydrogenlyase complex in CDP1 included four formate hydrogenlyase subunits (3, 5, 6, and 7; six coding sequences), five hydrogenase-4 subunits (hydrogenase 4 components A, C, D, E, and F; five coding sequences), and a formate hydrogenlyase transcriptional activator. Formate can be produced as a major fermentation product of microbes, such as the human gut microbiota. For example, formate is one of the major fermentation products of several dominant species of human colonic bacteria, including Eubacterium rectale, Roseburia inulinivorans, Eubacterium hallii, and Faecalibacterium prausnitzii (29). One of the genes for formate production encodes *S*-formylglutathione hydrolase (K01070), and in the amphipod gut metagenomes, a total of 14 *S*-formylglutathione hydrolase-encoding genes were identified.

**TABLE 2:** Comparison of the numbers of formate hydrogenlyase and hydrogenase-encoding genes in the CDP1 and reference genomes

Gene product or parameter	*Psychromonas* sp. strain CDP1	P. hadalis	P. ossibalaenae	P. aquimarina	P. arctica
Formate hydrogenlyase subunit 3	1	0	0	0	0
Formate hydrogenlyase subunit 5	3	0	0	0	0
Formate hydrogenlyase subunit 6	1	0	0	0	0
Formate hydrogenlyase subunit 7	1	0	0	0	0
Formate hydrogenlyase transcriptional activator	1	0	0	0	0
Hydrogenase-4 component A	1	0	0	0	0
Hydrogenase-4 component C	1	0	0	0	0
Hydrogenase-4 component D	1	0	0	0	0
Hydrogenase-4 component E	1	0	0	0	0
Hydrogenase-4 component F	1	0	0	0	0
Subtotal	12	0	0	0	0

## DISCUSSION

Gut metagenomes of H. gigas from two locations have dominant taxa in common, suggesting the existence of a specialized gut microbiota. The “*Candidatus* Hepatoplasma,” *Burkholderia*, and *Methanosarcina* bacteria identified in this study have also been reported to be symbionts of terrestrial isopods (30), bean bugs (31), and termites (32), respectively. The presence of these microbial groups points to a specific association between the gut microbiota and the H. gigas host. Functional classification suggests the overrepresentation of carbohydrate, amino acid, fatty acid, lipid, and protein metabolic genes, implying heterotrophic utilization of a variety of carbon sources. A number of genes involved in the biosynthesis of cofactors and vitamins are also present, suggesting the potential to maintain a mutualistic relationship with other microbial groups or the amphipod host.

While different microbial groups were found in the H. gigas gut, genomic analyses in the present study focused on *Psychromonas* because this is the dominant genus in a majority of the hadal H. gigas specimens examined and the extracted genomes of other microbial populations are more fragmented. Only one *Psychromonas* strain is present in each gut, and *Psychromonas* strains from different H. gigas individuals are very closely related members, suggesting a specific association between *Psychromonas* bacteria and their host. This specificity is, to some extent, consistent with the stability of invertebrate-associated microbial bacteria, such as *Nitrospira* in sponges (33). Specificity of host-microbe association can be explained by both vertical (microbes pass from the parents to the offspring of the hosts but are never present in external environments) and horizontal (the host acquires specific microbes from the external environment) mechanisms of transmission (33). However, the exact mechanisms and time scales used by H. gigas to preserve this remarkably stable association with *Psychromonas* strains remain unknown. One important feature of *Psychromonas* sp. strain CDP1 from an amphipod gut is genome reduction, consistent with the hypothesis that bacteria in some ecosystems are subject to strong selection to minimize the material costs of growth (34). Genome reduction is observed in many bacterial symbionts of invertebrates (reviewed in reference 35), such as those from deep-sea sponges (36). For example, compared with their free-living relatives, endosymbiotic sulfur-oxidizing bacteria of sponges have lost genes for carbohydrate nutrient utilization, including those for metabolism of saccharides, organic acids, and sugar alcohols (36). One possible explanation for genome reduction of hosted microbes is that selection has favored a smaller genome in pursuit of growth efficiency or competitiveness within the host (37). The genome reduction in CDP1 may reflect its long-term association with the H. gigas host.

The small genome of CDP1 is the result of a shortening of intergenic regions and gene deletion. One consequence of gene deletion is complete removal of the *torCAD* operon, which reduces TMAO to TMA to produce energy in bacteria (e.g., *Escherichia* and *Shewanella*). In this way, TMAO in bacterial cells acts as an electron acceptor during anaerobic respiration (38, 39). TMAO has also been found to enhance protein folding and to counteract hydrostatic pressure as a “piezolyte” (40) in both microbes and animals, and its abundance correlates positively with depth in deep-sea fish and invertebrates (41–43). The versatility of TMAO raises a possible conflict between its two functions in that when TMAO is removed by reduction to TMA during respiration, its piezolyte function will be less effective. The absence of the *torCAD* operon in the CDP1 genome suggests that the piezolyte function of TMAO (perhaps benefiting both the host and the symbiont) is more important than its function in respiration. That is, deletion of the *tor* operon leads to the consequence that TMAO can be accumulated to function as a piezolyte. Therefore, deletion of the *tor* system in CDP1 could be explained by the hydrostatic stress response of the host and the gut microbiota.

Equally important is the fact that the small genome of CDP1 can also be attributed to a lack of certain carbohydrate metabolism genes, compared to its relatives. The smaller number of genes participating in the utilization of starch, d-gluconate, lactose, and galactose monosaccharides in CDP1 than in the free-living references may reflect its specialized diet within the amphipod gut. Consistent with this scenario, a previous study demonstrated that the deletion of genes for degradation of carbon that can be obtained from the host appears to account for the gene reduction in a bumblebee gut bacterium (a Bombus impatiens gammaproteobacterium named *BiG*) (44). *BiG* contains a majority of the genes in central metabolic pathways, including glycolysis, gluconeogenesis, and the full pentose phosphate pathway, but has lost several genes used in the biosynthesis of branched-chain amino acids (44).

Despite having a small genome, CDP1 possesses a complete formate hydrogenlyase complex. The formate hydrogenlyase complex is used for anaerobic respiration in bacteria, decomposing formate to CO_2_ and H_2_ (45). This complex is expressed only in the absence of exogenous electron acceptors and is repressed by electron acceptors like oxygen and nitrate (46). In shallow-water reference strains, TMAO can serve as an exogenous electron acceptor and be reduced to TMA by the Tor system; however, in the CDP1 genome, the formate hydrogenlyase complex is likely to be employed for anaerobic respiration and energy production because TMAO cannot serve as an electron acceptor because of the absence of the *torCAD* operon. These results imply that CDP1 has developed adaptive strategies for a lifestyle within the gut of H. gigas living in the Mariana Trench through a long history of coevolution.

## MATERIALS AND METHODS

### Sample collection and extraction of DNA.

Amphipod H. gigas samples were collected from the Challenger Deep of the Mariana Trench (11°22′11ʺN, 142°35′25ʺE) at a depth of 10,929 m, as measured with a calibrated RBR 10,000-m pressure sensor, by the lander vehicle during the cruise of the Schmidt Ocean Institute. The lander was deployed on 18 December 2014 and recovered after 20 h. The amphipods were transferred to the cold room at 4°C after being brought to the deck and stored in Falcon tubes. The samples for DNA extraction were then frozen at −80°C in DNA extraction buffer within 1 h, and samples for microscopic observation were kept at 4°C. The Sirena Deep (12°30′27ʺN, 144°67′38ʺE) H. gigas samples were collected at a depth of 7,929 m (note that this is not the deepest point of the Sirena Deep). A free-fall trap lander (47) was deployed on 20 November 2014 and recovered after 28 h. These samples were obtained during the Schmidt Ocean Institute-funded RV *Falkor* cruises to the Marina Trench.

### DNA extraction, metagenomic sequencing, and analysis.

After dissection of the 11 amphipod individuals, DNA was extracted from the guts with the AllPrep DNA/RNA minikit (Qiagen, Hilden, Germany). The quality and quantity of the DNA were evaluated with a UV-Vis spectrophotometer (NanoDrop 2000; Thermo) and by agarose gel electrophoresis. The metagenomic DNA was sequenced with an Illumina HiSeq 2500 platform at Novogene Bioinformatics Technology Co., Ltd. (Beijing, China). The NGS QC Toolkit (version 2.3.3) (48) was used for quality control of the raw metagenomic reads (2 × 125 bp). For bases with a quality score of <20, the raw reads were trimmed from the 3′ end and those with a length of <70 after trimming were removed. Reads with an average quality score of <20 and with sequencing adaptors were also removed by filtering. The paired-end reads were assembled with MegaHit (version 1.0.2) (49) by using kmer values of 21 to 111 (step size, 10). The parameters used were --kmin-1pass (1pass mode to build SdBG of k_min) and --mem-flag 2. The coverage of the contigs was calculated with Bowtie 2 (50). The gene coding sequences within the assembled contigs were predicted with Prodigal (version 2.60) (51). The taxonomic affiliation of the proteins was analyzed by performing a BLASTP search (50 maximum hits, E value of <1e-5) of the NCBI NR database (2017 version) and using MEGAN2 (52) by the lowest common ancestor (LCA) method (minimum score, 110; top percentage, 10; LCA percentage, 80). Taxonomic composition of the metagenome was determined by integrating the taxonomic affiliation and read coverage of the protein sequences. Functional classification of the proteins derived from the metagenomes was conducted by a search of the SEED database (53) implemented in MEGAN2. CRISPRs were identified by using the Linux version of the CRT software (54).

### RNA extraction, cDNA synthesis, metatranscriptomic sequencing, and analysis.

RNA was extracted from the midgut and hindgut of one of the amphipods from the Challenger Deep with the AllPrep DNA/RNA minikit. To generate sufficient non-rRNA sequences for library construction and sequencing, the RNA samples were subjected to cDNA synthesis and amplification with the Ovation Single-Cell RNA-Seq System (NuGEN Technologies, Inc.) in accordance with the manufacturer’s instructions. The cDNA products were sequenced with the Illumina HiSeq 2500 platform at Novogene. The metatranscriptomic reads were mapped to the genome coding sequences with Bowtie 2 (50), the mapped genes were annotated with the carbohydrate-active enzyme (CAZy) database, and then the number of reads per kilobase per million mapped reads was calculated.

### FISH experiment.

A FISH experiment was performed as described previously (55). An amphipod individual was fixed in 4% formaldehyde (Merck, Germany) buffered with 1× phosphate-buffered saline (PBS) at 4°C overnight. The sample was then washed three times with PBS before dehydration by sequential washing in 70, 80, 95, and 100% ethanol (40 min each). Xylene was applied three times to replace the ethanol (40 min each), followed by three washes with paraffin (40 min each). The sample was then embedded in paraffin and cooled to room temperature. Sections (5 µm) were cut with a microtome (Leica, Germany) and placed on a drop of distilled water on 0.01% poly-l-lysine-coated coverslips, followed by heating at 50°C for 10 min. The coverslips were dried in a chemical hood overnight. The sections were deparaffinized with xylene before rehydration by serial washing in 95, 80, and 70% ethanol. Cy3-labeled probes EUB338 (5′ GCTGCCTCCCGTAGGAGT 3′) (56) and PSY429 (5′ GCGCCTCAGTGTCAGTCTTT 3′), respectively, were used to stain all of the bacterial and *Psychromonas* cells in the sections. PSY429 was designed in the present study, and the sequence of this probe is part of the *Psychromonas* 16S rRNA gene identified in the metatranscriptomes with the Meta-RNA program (57). The probes and samples were incubated in hybridization buffer containing 30% formamide (probe concentration, 5 ng/µl) at 46°C for 90 min. A parallel negative control with an unrelated and Cy3-labeled probe (5′ TATTGGTCCAAGAAGTCGCC 3′) (55) was exposed to the same conditions. The sections were washed twice with washing buffer at 48°C (20 min each) and then stained for 3 min with 4ʹ,6-diamidino-2-phenylindole (DAPI; 5 ng/µl) and washed for 5 min with 80% ethanol. After air drying, the sections were mounted with CitiFluor AF4 mounting medium (CitiFluor Ltd.) and observed under a fluorescence microscope (Olympus BX51).

### Genome assembly and gap closing.

The *Psychromonas* draft genome was binned as described previously (58, 59) and used in our recent deep-sea microbiome studies (55, 60). The Illumina reads of two different samples from the midgut and hindgut of one amphipod individual from the Challenger Deep were mapped onto the contigs with Bowtie 2 (50), and the sequencing coverage of the contigs was calculated with SAMtools (version 0.1.19) (61). The GC content and tetranucleotide frequency of the contigs were calculated with Perl scripts (calc.gc.pl and calc.kmerfreq.pl, available at https://github.com/MadsAlbertsen/multi-metagenome/tree/master/R.data.generation). Protein-coding sequences were identified with Prodigal (version 2.60) (51) and conserved single-copy genes were identified by hmmsearch of the Pfam database (62). The sequencing coverage for the contigs in two metagenomes (Fig. S4), GC content and tetranucleotide frequency of the contigs, and taxonomic affiliation of the single-copy genes derived from the contigs were then imported into the RStudio platform to classify these contigs into different genomes, and the contigs belonging to the *Psychromonas* strain were extracted (see the supplemental material for reference 58 for details). Completeness of the draft genome was estimated through analysis of conserved single-copy genes as described previously to calculate genome bin completeness (63). Contamination of the extracted draft genome was evaluated with CheckM (version 1.0.5) (64). Figure S4 also shows that most of the assembled contigs belonged to *Psychromonas*, making it difficult to bin the genomes of other microbes.

10.1128/mSystems.00009-18.4 FIG S4  The sequencing coverage of the assembled contigs in two metagenomes. Each circle represents a contig assembled from metagenomic reads. The *x* and *y* axes indicate the coverage for these contigs in the two metagenomes used for genome binning. Circle size indicates contig length. The colors represent taxonomic affiliations at the phylum level for the microbe-derived contigs (e.g., *Proteobacteria*), while the black circles are contigs belonging to the amphipod genome. Download FIG S4, DOCX file, 0.3 MB. Copyright © 2018 Zhang et al. 2018 Zhang et al. This content is distributed under the terms of the Creative Commons Attribution 4.0 International license.

Next, reassembly of the draft genome was initialized by mapping reads of the six Challenger Deep metagenomes (CD1-midgut, CD1-hindgut, CD2, CD3, CD4, and CD5) to the draft genome with the GenomeMapper software (65). The mapped reads were extracted from the metagenomes, pooled, and then reassembled with SPAdes (66) by using the parameters --trusted-contigs draft-genome-bin. The reassembly step generated 11 contigs, which were then aligned with the complete genomes of other *Psychromonas* strains to put the contigs into the correct orientation. PCRs were carried out with primer pairs flanking two adjacent contigs in the Phusion High-Fidelity DNA polymerase (Finnzymes Oy, Espoo, Finland) system (0.4 U of polymerase, 1× HF reaction buffer, 0.1 mM each bar-coded primer, 0.6 µl of dimethyl sulfoxide, 0.2 mM deoxynucleoside triphosphates, 10 ng of purified DNA template), and metagenomic DNA from the Challenger Deep amphipod was used as templates. The detailed PCR steps were initial denaturation at 98°C for 30 s; 26 cycles of 98°C for 10 s, 60°C for 10 s, and 72°C for 60 s; and a final extension at 72°C for 5 min. The PCR products were purified with the Tiangen DNA kit (Tiangen Biotech, Beijing, China), and then libraries were constructed with the pMD18-T cloning kit (TaKaRa, Dalian, China). The detailed steps of gene cloning were described previously (67). The libraries were sequenced with the Sanger sequencing platform at BGI Co., Ltd. (Shenzhen, China). The output of the sequencing was manually linked to the draft genome of CDP1, and after sequencing of 11 libraries, eight gaps were successfully closed, resulting in a nearly complete genome composed of three contigs. The genomic features were prepared with CGView (68). The ANIs between CDP1 and other *Psychromonas* genomes were calculated with a recently developed online pipeline (http://www.ezbiocloud.net/tools/ani) (69).

### Phylogenetic analysis based on marker genes.

Marker genes were identified by AMPHORA2 (70), which documents 31 marker genes selected on the basis of the construction of a genome tree of 578 bacterial species. We searched for these 31 marker genes by hmmsearch of the Pfam database (62) and found 27 marker genes common to the CDP1 genome and all of the reference genomes. The 27 genes included those for ribosomal proteins L3 and L20; the DNA-directed RNA polymerase beta subunit; ribosomal protein S2; translation elongation factors Ts and 3; ribosomal proteins L14, L27, L2, L19, S13, L6P L9E, L16 L10E, and L7 L12; transcription elongation factor; ribosomal protein L1; 3-phosphoglycerate kinase; ribosomal proteins S11, L11, L4, L5, S5, S19, and S3; DNA primase; and tmRNA-binding protein. For phylogenetic analysis, the 27 marker genes were aligned and concatenated to generate an alignment length of 5,552 amino acids. The aligned sequences were imported into MEGA (version 7.0.21) (71) to construct a maximum-likelihood tree by using the Jones-Taylor-Thornton model (this model was selected on the basis of the test by ProtTest [72]) and a bootstrap replication number of 500.

### Phylogenetic analysis based on 16S rRNA genes.

*Psychromonas* 16S rRNA genes were recovered from the gut metagenomes with the Meta-RNA program (57). Alignment of nearly full-length (>1,400 bp) 16S rRNA genes was conducted in MEGA (version 7.0.21) (71) with the MUSCLE algorithm by using a gap open penalty of −50, the unweighted pair group method, and a minimum diagonal length of 24. The alignment length was 1,545 bp. A maximum-likelihood tree of 16S rRNA genes was constructed with MEGA (version 7.0.21) (71) by using the Tamura-Nei model and the nearest-neighbor interchange method with 500 bootstrap replicates.

### Genome annotation.

The gene coding sequences of the draft *Psychromonas* genome and all of the other reference genomes were predicted with Prodigal (version 2.60) (51). Genome annotation was performed by performing a BLASTP search of the COG database (73). Metabolic pathways were reconstructed by performing a BLASTP search of the KEGG database (74). A functional hierarchical comparison with the reference genomes was performed with the SEED functional classification system (53) implemented in MEGAN (version 6.7.8) (52), where the results of a BLASTP search of the NCBI NR database (version updated in 2017) with 50 maximum hits and an E value of <1e-5 were used as input files.

### Statistical analysis for gene abundance comparison.

The Mann-Whitney U test, a nonparametric test that allows two groups to be compared without normally distributed values, was performed with the online calculator (75).

### Data availability.

The metagenomic and metatranscriptomic data obtained in this study have been submitted to the Sequence Read Archive database under accession numbers SRP071106 and SRP071109, respectively. The genome sequences of the new *Psychromonas* strain have been deposited in DDBJ/EMBL/GenBank under accession number NDFH00000000.

## References

[1] Wolff T 1959 The hadal community, an introduction. Deep Sea Res 6:95–124. doi:10.1016/0146-6313(59)90063-2.

[2] Lauro FM, Bartlett DH 2008 Prokaryotic lifestyles in deep sea habitats. Extremophiles 12:15–25. doi:10.1007/s00792-006-0059-5. 17225926

[3] Jamieson AJ, Fujii T, Solan M, Matsumoto AK, Bagley PM, Priede IG 2009 First findings of decapod crustacea in the hadal zone. Deep Sea Res Part 1 Oceanogr Res Pap 56:641–647. doi:10.1016/j.dsr.2008.11.003.

[4] Nunoura T, Takaki Y, Hirai M, Shimamura S, Makabe A, Koide O, Kikuchi T, Miyazaki J, Koba K, Yoshida N, Sunamura M, Takai K 2015 Hadal biosphere: insight into the microbial ecosystem in the deepest ocean on Earth. Proc Natl Acad Sci U S A 112:E1230–E1236. doi:10.1073/pnas.1421816112. PMC4371994 25713387

[5] Tarn J, Peoples LM, Hardy K, Cameron J, Bartlett DH 2016 Identification of free-living and particle-associated microbial communities present in hadal regions of the Mariana Trench. Front Microbiol 7:665. doi:10.3389/fmicb.2016.00665. 27242695 PMC4860528

[6] Glud RN, Wenzhöfer F, Middelboe M, Oguri K, Turnewitsch R, Canfield DE, Kitazato H 2013 High rates of microbial carbon turnover in sediments in the deepest oceanic trench on Earth. Nat Geosci 6:284–288. doi:10.1038/ngeo1773.

[7] Li M, Wang B, Zhang M, Rantalainen M, Wang S, Zhou H, Zhang Y, Shen J, Pang X, Zhang M, Wei H, Chen Y, Lu H, Zuo J, Su M, Qiu Y, Jia W, Xiao C, Smith LM, Yang S, Holmes E, Tang H, Zhao G, Nicholson JK, Li L, Zhao L 2008 Symbiotic gut microbes modulate human metabolic phenotypes. Proc Natl Acad Sci U S A 105:2117–2122. doi:10.1073/pnas.0712038105. 18252821 PMC2538887

[8] Clemente JC, Ursell LK, Parfrey LW, Knight R 2012 The impact of the gut microbiota on human health: an integrative view. Cell 148:1258–1270. doi:10.1016/j.cell.2012.01.035. 22424233 PMC5050011

[9] Dugas LR, Fuller M, Gilbert J, Layden BT 2016 The obese gut microbiome across the epidemiologic transition. Emerg Themes Epidemiol 13:2. doi:10.1186/s12982-015-0044-5. 26759600 PMC4710045

[10] Clarke G, Stilling RM, Kennedy PJ, Stanton C, Cryan JF, Dinan TG 2014 Minireview: gut microbiota: the neglected endocrine organ. Mol Endocrinol 28:1221–1238. doi:10.1210/me.2014-1108. 24892638 PMC5414803

[11] Zhang M, Sun Y, Liu Y, Qiao F, Chen L, Liu WT, Du Z, Li E 2016 Response of gut microbiota to salinity change in two euryhaline aquatic animals with reverse salinity preference. Aquaculture 454:72–80. doi:10.1016/j.aquaculture.2015.12.014.

[12] Thurston MH 1979 Scavenging abyssal amphipods from the North-East Atlantic Ocean. Mar Biol 51:55–68. doi:10.1007/BF00389031.

[13] Hessler RR, Isaacs JD, Mills EL 1972 Giant amphipod from the abyssal Pacific Ocean. Science 175:636–637. doi:10.1126/science.175.4022.636. 17808804

[14] Shulenberger E, Barnard JL 1976 Amphipods from an abyssal trap set in the North Pacific Gyre. Crustaceana 31:241–258. doi:10.1163/156854076X00035.

[15] Eustace RM, Kilgallen NM, Lacey NC, Jamieson AJ 2013 Population structure of the hadal amphipod *Hirondellea gigas* from the Izu-Bonin Trench. J Crustacean Biol 33:793–801. doi:10.1163/1937240X-00002193.

[16] Jamieson AJ, Kilgallen NM, Rowden AA, Fujii T, Horton T, Lörz A-N, Kitazawa K, Priede IG 2011 Bait-attending fauna of the Kermadec Trench, SW Pacific Ocean: evidence for an ecotone across the abyssal-hadal transition zone. Deep Sea Res Part 1 Oceanogr Res Pap 58:49–62. doi:10.1016/j.dsr.2010.11.003.

[17] Fujii T, Kilgallen NM, Rowden AA, Jamieson AJ 2013 Amphipod community structure across abyssal to hadal depths in the Peru-Chile and the Kermadec Trenches. Mar Ecol Prog Ser 492:125–138. doi:10.3354/meps10489.

[18] Lacey NC, Rowden AA, Clark MR, Kilgallen NM, Linley TD, Mayor DJ, Jamieson AJ 2016 Community structure and diversity of scavenging amphipods from bathyal to hadal depths in three South Pacific trenches. Deep Sea Res Part 1 Oceanogr Res Pap 111:121–137. doi:10.1016/j.dsr.2016.02.014.

[19] Hessler RR, Ingram CL, Aristides Yayanos AA, Burnett BR 1978 Scavenging amphipods from the floor of the Philippine Trench. Deep Sea Res 25:1029–1047. doi:10.1016/0146-6291(78)90585-4.

[20] Kobayashi H, Hatada Y, Tsubouchi T, Nagahama T, Takami H 2012 The hadal amphipod *Hirondellea gigas* possessing a unique cellulase for digesting wooden debris buried in the deepest seafloor. PLoS One 7:e42727. doi:10.1371/journal.pone.0042727. 22905166 PMC3419748

[21] Nogi Y, Hosoya S, Kato C, Horikoshi K 2007 *Psychromonas hadalis* sp. nov., a novel piezophilic bacterium isolated from the bottom of the Japan Trench. Int J Syst Evol Microbiol 57:1360–1364. doi:10.1099/ijs.0.64933-0. 17551059

[22] Miyazaki M, Nogi Y, Fujiwara Y, Horikoshi K 2008 *Psychromonas japonica* sp. nov., *Psychromonas aquimarina* sp. nov., *Psychromonas macrocephali* sp. nov. and *Psychromonas ossibalaenae* sp. nov., psychrotrophic bacteria isolated from sediment adjacent to sperm whale carcasses off Kagoshima, Japan. Int J Syst Evol Microbiol 58:1709–1714. doi:10.1099/ijs.0.65744-0. 18599721

[23] Groudieva T, Grote R, Antranikian G 2003 *Psychromonas arctica* sp. nov., a novel psychrotolerant, biofilm-forming bacterium isolated from Spitzbergen. Int J Syst Evol Microbiol 53:539–545. doi:10.1099/ijs.0.02182-0. 12710624

[24] Lan Y, Sun J, Bartlett DH, Rouse GW, Tabata HG, Qian P 2016 The deepest mitochondrial genome sequenced from Mariana Trench *Hirondellea gigas* (Amphipoda). Mitochondrial DNA B Resour 1:802–803. doi:10.1080/23802359.2016.1214549. PMC7799506 33473633

[25] Orsi WD, Edgcomb VP, Christman GD, Biddle JF 2013 Gene expression in the deep biosphere. Nature 499:205–208. doi:10.1038/nature12230. 23760485

[26] Méjean V, Iobbi-Nivol C, Lepelletier M, Giordano G, Chippaux M, Pascal MC 1994 TMAO anaerobic respiration in *Escherichia coli*-involvement of the *tor* operon. Mol Microbiol 11:1169–1179. doi:10.1111/j.1365-2958.1994.tb00393.x. 8022286

[27] Gon S, Jourlin-Castelli C, Théraulaz L, Méjean V 2001 An unsuspected autoregulatory pathway involving apocytochrome TorC and sensor TorS in *Escherichia coli*. Proc Natl Acad Sci U S A 98:11615–11620. doi:10.1073/pnas.211330598. 11562502 PMC58778

[28] Andrade SL, Einsle O 2007 The Amt/Mep/Rh family of ammonium transport proteins. Mol Membr Biol 24:357–365. doi:10.1080/09687680701388423. 17710640

[29] Flint HJ, Duncan SH, Scott KP, Louis P 2015 Links between diet, gut microbiota composition and gut metabolism. Proc Nutr Soc 74:13–22. doi:10.1017/S0029665114001463. 25268552

[30] Wang Y, Stingl U, Anton-Erxleben F, Geisler S, Brune A, Zimmer M 2004 “*Candidatus* Hepatoplasma crinochetorum,” a new, stalk-forming lineage of Mollicutes colonizing the midgut glands of a terrestrial isopod. Appl Environ Microbiol 70:6166–6172. doi:10.1128/AEM.70.10.6166-6172.2004. 15466563 PMC522098

[31] Kim JK, Lee JB, Huh YR, Am Jang HA, Kim CH, Yoo JW, Lee BL 2015 *Burkholderia* gut symbionts enhance the innate immunity of host *Riptortus pedestris*. Dev Comp Immunol 53:265–269. doi:10.1016/j.dci.2015.07.006. 26164198

[32] Kavitha S, Gopinath LR, Christy PM 2014 Isolation of methanogens from termite gut and its role in biogas production by using poultry waste. Int J Plant Anim Environ Sci 4:281–286.

[33] Reveillaud J, Maignien L, Murat Eren AM, Huber JA, Apprill A, Sogin ML, Vanreusel A 2014 Host-specificity among abundant and rare taxa in the sponge microbiome. ISME J 8:1198–1209. doi:10.1038/ismej.2013.227. 24401862 PMC4030224

[34] Giovannoni SJ, Tripp HJ, Givan S, Podar M, Vergin KL, Baptista D, Bibbs L, Eads J, Richardson TH, Noordewier M, Rappé MS, Short JM, Carrington JC, Mathur EJ 2005 Genome streamlining in a cosmopolitan oceanic bacterium. Science 309:1242–1245. doi:10.1126/science.1114057. 16109880

[35] McCutcheon JP, Moran NA 2011 Extreme genome reduction in symbiotic bacteria. Nat Rev Microbiol 10:13–26. doi:10.1038/nrmicro2670. 22064560

[36] Tian RM, Zhang W, Cai L, Wong YH, Ding W, Qian PY 2017 Genome reduction and microbe-host interactions drive adaptation of a sulfur-oxidizing bacterium associated with a cold seep sponge. mSystems 2:e00184-16. doi:10.1128/mSystems.00184-16. 28345060 PMC5361782

[37] Moran NA 2002 Microbial minimalism: genome reduction in bacterial pathogens. Cell 108:583–586. doi:10.1016/S0092-8674(02)00665-7. 11893328

[38] Gillett MB, Suko JR, Santoso FO, Yancey PH 1997 Elevated levels of trimethylamine oxide in muscles of deep-sea gadiform teleosts: a high-pressure adaptation? J Exp Zool 279:386–391. doi:10.1002/(SICI)1097-010X(19971101)279:4<386::AID-JEZ8>3.0.CO;2-K.

[39] Kelly RH, Yancey PH 1999 High contents of trimethylamine oxide correlating with depth in deep-sea teleost fishes, skates, and decapod crustaceans. Biol Bull 196:18–25. doi:10.2307/1543162. 25575382

[40] Yancey PH, Gerringer ME, Drazen JC, Rowden AA, Jamieson AJ 2014 Marine fish may be biochemically constrained from inhabiting the deepest ocean depths. Proc Natl Acad Sci U S A 111:4461–4465. doi:10.1073/pnas.1322003111. 24591588 PMC3970477

[41] Barrett EL, Kwan HS 1985 Bacterial reduction of trimethylamine oxide. Annu Rev Microbiol 39:131–149. doi:10.1146/annurev.mi.39.100185.001023. 3904597

[42] Dos Santos JP, Iobbi-Nivol C, Couillault C, Giordano G, Méjean V 1998 Molecular analysis of the trimethylamine N-oxide (TMAO) reductase respiratory system from a *Shewanella* species. J Mol Biol 284:421–433. doi:10.1006/jmbi.1998.2155. 9813127

[43] Martin DD, Bartlett DH, Roberts MF 2002 Solute accumulation in the deep-sea bacterium *Photobacterium profundum*. Extremophiles 6:507–514. doi:10.1007/s00792-002-0288-1. 12486460

[44] Martinson VG, Magoc T, Koch H, Salzberg SL, Moran NA 2014 Genomic features of a bumble bee symbiont reflect its host environment. Appl Environ Microbiol 80:3793–3803. doi:10.1128/AEM.00322-14. 24747890 PMC4054214

[45] McDowall JS, Murphy BJ, Haumann M, Palmer T, Armstrong FA, Sargent F 2014 Bacterial formate hydrogenlyase complex. Proc Natl Acad Sci U S A 111:E3948–E3956. doi:10.1073/pnas.1407927111. 25157147 PMC4183296

[46] Axley MJ, Grahame DA, Stadtman TC 1990 *Escherichia coli* formate-hydrogen lyase. Purification and properties of the selenium-dependent formate dehydrogenase component. J Biol Chem 265:18213–18218. 2211698

[47] Linley TD, Gerringer ME, Yancey PH, Drazen JC, Weinstock CL, Jamieson AJ 2016 Fishes of the hadal zone including new species, in situ observations and depth records of hadal snailfishes. Deep Sea Res 114:99–110. doi:10.1016/j.dsr.2016.05.003.

[48] Patel RK, Jain M 2012 NGS QC toolkit: a toolkit for quality control of next generation sequencing data. PLoS One 7:e30619. doi:10.1371/journal.pone.0030619. 22312429 PMC3270013

[49] Li DH, Liu CM, Luo RB, Sadakane K, Lam TW 2015 MEGAHIT: an ultra-fast single-node solution for large and complex metagenomics assembly via succinct de Bruijn graph. Bioinformatics 31:1674–1676. doi:10.1093/bioinformatics/btv033. 25609793

[50] Langmead B, Salzberg SL 2012 Fast gapped-read alignment with Bowtie 2. Nat Methods 9:357–359. doi:10.1038/nmeth.1923. 22388286 PMC3322381

[51] Hyatt D, Chen GL, LoCascio PF, Land ML, Larimer FW, Hauser LJ 2010 Prodigal: prokaryotic gene recognition and translation initiation site identification. BMC Bioinformatics 11:119. doi:10.1186/1471-2105-11-119. 20211023 PMC2848648

[52] Huson DH, Beier S, Flade I, Górska A, El-Hadidi M, Mitra S, Ruscheweyh HJ, Tappu R 2016 MEGAN community edition-interactive exploration and analysis of large-scale microbiome sequencing data. PLoS Comput Biol 12:e1004957. doi:10.1371/journal.pcbi.1004957. 27327495 PMC4915700

[53] Overbeek R, Begley T, Butler RM, Choudhuri JV, Chuang HY, Cohoon M, de Crécy-Lagard V, Diaz N, Disz T, Edwards R, Fonstein M, Frank ED, Gerdes S, Glass EM, Goesmann A, Hanson A, Iwata-Reuyl D, Jensen R, Jamshidi N, Krause L, Kubal M, Larsen N, Linke B, McHardy AC, Meyer F, Neuweger H, Olsen G, Olson R, Osterman A, Portnoy V, Pusch GD, Rodionov DA, Rückert C, Steiner J, Stevens R, Thiele I, Vassieva O, Ye Y, Zagnitko O, Vonstein V 2005 The subsystems approach to genome annotation and its use in the project to annotate 1000 genomes. Nucleic Acids Res 33:5691–5702. doi:10.1093/nar/gki866. 16214803 PMC1251668

[54] Bland C, Ramsey TL, Sabree F, Lowe M, Brown K, Kyrpides NC, Hugenholtz P 2007 CRISPR recognition tool (CRT): a tool for automatic detection of clustered regularly interspaced palindromic repeats. BMC Bioinformatics 8:209. doi:10.1186/1471-2105-8-209. 17577412 PMC1924867

[55] Tian RM, Wang Y, Bougouffa S, Gao ZM, Cai L, Bajic V, Qian PY 2014 Genomic analysis reveals versatile heterotrophic capacity of a potentially symbiotic sulfur-oxidizing bacterium in sponge. Environ Microbiol 16:3548–3561. doi:10.1111/1462-2920.12586. 25088944

[56] Amann RI, Binder BJ, Olson RJ, Chisholm SW, Devereux R, Stahl DA 1990 Combination of 16S rRNA-targeted oligonucleotide probes with flow cytometry for analyzing mixed microbial populations. Appl Environ Microbiol 56:1919–1925. 2200342 10.1128/aem.56.6.1919-1925.1990 PMC184531

[57] Huang Y, Gilna P, Li W 2009 Identification of ribosomal RNA genes in metagenomic fragments. Bioinformatics 25:1338–1340. doi:10.1093/bioinformatics/btp161. 19346323 PMC2677747

[58] Albertsen M, Hugenholtz P, Skarshewski A, Nielsen KL, Tyson GW, Nielsen PH 2013 Genome sequences of rare, uncultured bacteria obtained by differential coverage binning of multiple metagenomes. Nat Biotechnol 31:533–538. doi:10.1038/nbt.2579. 23707974

[59] Sangwan N, Xia F, Gilbert JA 2016 Recovering complete and draft population genomes from metagenome datasets. Microbiome 4:8. doi:10.1186/s40168-016-0154-5. 26951112 PMC4782286

[60] Zhang W, Sun J, Cao H, Tian R, Cai L, Ding W, Qian PY 2016 Post-translational modifications are enriched within protein functional groups important to bacterial adaptation within a deep-sea hydrothermal vent environment. Microbiome 4:49. doi:10.1186/s40168-016-0194-x. 27600525 PMC5012046

[61] Li H, Handsaker B, Wysoker A, Fennell T, Ruan J, Homer N, Marth G, Abecasis G, Durbin R, 1000 Genome Project Data Processing Subgroup 2009 The sequence alignment/map format and SAMtools. Bioinformatics 25:2078–2079. doi:10.1093/bioinformatics/btp352. 19505943 PMC2723002

[62] Bateman A, Coin L, Durbin R, Finn RD, Hollich V, Griffiths-Jones S, Khanna A, Marshall M, Moxon S, Sonnhammer EL, Studholme DJ, Yeats C, Eddy SR 2004 The Pfam protein families database. Nucleic Acids Res 32:D138–D141. doi:10.1093/nar/gkh121. 14681378 PMC308855

[63] Rinke C, Schwientek P, Sczyrba A, Ivanova NN, Anderson IJ, Cheng JF, Darling A, Malfatti S, Swan BK, Gies EA, Dodsworth JA, Hedlund BP, Tsiamis G, Sievert SM, Liu WT, Eisen JA, Hallam SJ, Kyrpides NC, Stepanauskas R, Rubin EM, Hugenholtz P, Woyke T 2013 Insights into the phylogeny and coding potential of microbial dark matter. Nature 499:431–437. doi:10.1038/nature12352. 23851394

[64] Parks DH, Imelfort M, Skennerton CT, Hugenholtz P, Tyson GW 2015 CheckM: assessing the quality of microbial genomes recovered from isolates, single cells, and metagenomes. Genome Res 25:1043–1055. doi:10.1101/gr.186072.114. 25977477 PMC4484387

[65] Schneeberger K, Hagmann J, Ossowski S, Warthmann N, Gesing S, Kohlbacher O, Weigel D 2009 Simultaneous alignment of short reads against multiple genomes. Genome Biol 10:R98. doi:10.1186/gb-2009-10-9-r98. 19761611 PMC2768987

[66] Bankevich A, Nurk S, Antipov D, Gurevich AA, Dvorkin M, Kulikov AS, Lesin VM, Nikolenko SI, Pham S, Prjibelski AD, Pyshkin AV, Sirotkin AV, Vyahhi N, Tesler G, Alekseyev MA, Pevzner PA 2012 SPAdes: a new genome assembly algorithm and its applications to single-cell sequencing. J Comput Biol 19:455–477. doi:10.1089/cmb.2012.0021. 22506599 PMC3342519

[67] Zhang W, Wang Y, Song Y, Wang T, Xu S, Peng Z, Lin X, Zhang L, Shen X 2013 A type VI secretion system regulated by OmpR in *Yersinia pseudotuberculosis* functions to maintain intracellular pH homeostasis. Environ Microbiol 15:557–569. doi:10.1111/1462-2920.12005. 23094603

[68] Stothard P, Wishart DS 2005 Circular genome visualization and exploration using CGView. Bioinformatics 21:537–539. doi:10.1093/bioinformatics/bti054. 15479716

[69] Yoon SH, Ha SM, Lim JM, Kwon SJ, Chun J 2017 A large-scale evaluation of algorithms to calculate average nucleotide identity. Antonie Leeuwenhoek 110:1281–1286. doi:10.1007/s10482-017-0844-4. 28204908

[70] Wu M, Scott AJ 2012 Phylogenomic analysis of bacterial and archaeal sequences with AMPHORA2. Bioinformatics 28:1033–1034. doi:10.1093/bioinformatics/bts079. 22332237

[71] Kumar S, Stecher G, Tamura K 2016 MEGA7: molecular evolutionary genetics analysis version 7.0 for bigger datasets. Mol Biol Evol 33:1870–1874. doi:10.1093/molbev/msw054. 27004904 PMC8210823

[72] Abascal F, Zardoya R, Posada D 2005 ProtTest: selection of best-fit models of protein evolution. Bioinformatics 21:2104–2105. doi:10.1093/bioinformatics/bti263. 15647292

[73] Galperin MY, Makarova KS, Wolf YI, Koonin EV 2015 Expanded microbial genome coverage and improved protein family annotation in the COG database. Nucleic Acids Res 43:D261–D269. doi:10.1093/nar/gku1223 25428365 PMC4383993

[74] Kanehisa M, Goto S 2000 KEGG: Kyoto encyclopedia of genes and genomes. Nucleic Acids Res 28:27–30. doi:10.1093/nar/28.1.27. 10592173 PMC102409

[75] McKnight PE, Najab J 2010 Mann-Whitney U test, p 960 *In* Weiner IB, Craighead WE (ed), Corsini encyclopedia of psychology, 4th ed, vol 3, M-Q Wiley, New York, NY.

