# Supplementary material for: Genome Reduction in Psychromonas Species within the Gut of an Amphipod from the Ocean’s Deepest Point
Source: mSystems. 2018 Apr 10;3(3):e00009-18. doi: 10.1128/mSystems.00009-18 (PMC5893861; doi:10.1128/mSystems.00009-18)
Supplement: TABLE S1 [file sys003182223st1.docx]

**Table S1**

|  | Total number of reads | | Percentage of HQ reads | | Total number of bases | | Total number of bases in HQ reads | | Total number of HQ bases in HQ reads | | Percentage of HQ bases in HQ reads | | Number of Primer/Adaptor contaminated HQ reads | | Total number of HQ filtered reads | | Percentage of HQ filtered reads |
| --- | --- | --- | --- | --- | --- | --- | --- | --- | --- | --- | --- | --- | --- | --- | --- | --- | --- |
| Metagenome |  | |  | |  | |  | |  | |  | |  | |  | |  |
| CD1-midgut-1 | 91,475,763 | | 92.41% | | 10,359,426,809 | | 9,573,661,206 | | 9,255,091,556 | | 96.67% | | 701 | | 84,534,245 | | 92.41% |
| CD1-midgut-2 | 91,475,763 | | 92.41% | | 10,359,426,809 | | 9,573,661,206 | | 9,109,546,763 | | 95.15% | | 10 | | 84,534,245 | | 92.41% |
| CD1-hindgut-1 | 97,613,112 | | 92.41% | | 11,059,710,176 | | 10,332,946,245 | | 10,001,849,121 | | 96.8% | | 446 | | 91,194,087 | | 93.42% |
| CD1-hindgut-2 | 97,613,112 | | 92.41% | | 11,059,710,176 | | 10,332,946,245 | | 9,882,515,697 | | 95.64% | | 18 | | 91,194,087 | | 93.42% |
| CD2-gut-1 | 46,087,114 | | 100.00% | | 6,913,067,100 | | 6,913,067,100 | | 6,765,759,288 | | 97.87% | | 8,965 | | 46,070,815 | | 99.96% |
| CD2-gut-2 | 46,087,114 | | 100.00% | | 6,913,067,100 | | 6,913,067,100 | | 6,398,088,723 | | 92.55% | | 7,473 | | 46,070,815 | | 99.96% |
| CD3-gut-1 | 40,186,496 | | 100.00% | | 6,027,974,400 | | 6,027,974,400 | | 5,874,848,846 | | 97.46% | | 12,751 | | 401,653,27 | | 99.95% |
| CD3-gut-2 | 40,186,496 | | 100.00% | | 6,027,974,400 | | 6,027,974,400 | | 5,659,513,182 | | 93.89% | | 8,634 | | 40,165,327 | | 99.95% |
| CD4-gut-1 | 39,491,900 | | 100.00% | | 5,923,785,000 | | 5,923,785,000 | | 5,736,921,023 | | 96.85% | | 8,426 | | 39,477,860 | | 99.96% |
| CD4-gut-2 | 39,491,900 | | 100.00% | | 5,923,785,000 | | 5,923,785,000 | | 5,552,793,699 | | 93.74% | | 5,735 | | 39,477,860 | | 99.96% |
| CD5-gut-1 | 33,456,840 | | 100.00% | | 5,018,526,000 | | 5,018,526,000 | | 4,877,721,323 | | 97.19% | | 8,581 | | 33,442,732 | | 99.96% |
| CD5-gut-2 | 33,456,840 | | 100.00% | | 5,018,526,000 | | 5,018,526,000 | | 4,659,745,125 | | 92.85% | | 5,651 | | 33,442,732 | | 99.96% |
| SD1-gut-1 | 39,403,070 | | 100.00% | | 5,910,460,500 | | 5,910,460,500 | | 5,827,949,369 | | 98.60% | | 10,581 | | 39,380,723 | | 99.94% |
| SD1-gut-2 | 39,403,070 | | 100.00% | | 5,910,460,500 | | 5,910,460,500 | | 5,697,689,631 | | 96.40% | | 11,858 | | 39,380,723 | | 99.94% |
| SD2-gut-1 | 46,087,114 | | 100.00% | | 6,913,067,100 | | 6,913,067,100 | | 6,765,759,288 | | 97.87% | | 8,965 | | 46,070,815 | | 99.96% |
| SD2-gut-2 | 46,087,114 | | 100.00% | | 6,913,067,100 | | 6,913,067,100 | | 6,398,088,723 | | 92.55% | | 7,473 | | 46,070,815 | | 99.96% |
| SD3-gut-1 | 58,535,538 | | 100.00% | | 8,780,330,700 | | 8,780,330,700 | | 8,638,369,957 | | 98.38% | | 11,690 | | 58,516,119 | | 99.97% |
| SD3-gut-2 | 58,535,538 | | 100.00% | | 8,780,330,700 | | 8,780,330,700 | | 8,344,443,610 | | 95.04% | | 7,821 | | 58,516,119 | | 99.97% |
| SD4-gut-1 | 21,951,119 | | 100.00% | | 3,292,667,850 | | 3,292,667,850 | | 3,212,431,022 | | 97.56% | | 16,129 | | 21,918,444 | | 99.85% |
| SD4-gut-2 | 21,951,119 | | 100.00% | | 3,292,667,850 | | 3,292,667,850 | | 3,056,850,079 | | 92.84% | | 17,598 | | 21,918,444 | | 99.85% |
| SD5-gut-1 | 39,941,312 | | 100.00% | | 5,991,196,800 | | 5,991,196,800 | | 5,874,299,227 | | 98.05% | | 5,570 | | 39,931,100 | | 99.97% |
| SD5-gut-2 | 39,941,312 | | 100.00% | | 5,991,196,800 | | 5,991,196,800 | | 5,569,077,851 | | 92.95% | | 4,678 | | 39,931,100 | | 99.97% |
| SD6-gut-1 | 36,203,022 | | 100.00% | | 5,430,453,300 | | 5,430,453,300 | | 5,322,812,767 | | 98.02% | | 8,723 | | 36,185,438 | | 99.95% |
| SD6-gut-2 | 36,203,022 | | 100.00% | | 543,0453,300 | | 5,430,453,300 | | 5,048,256,529 | | 92.96% | | 9,048 | | 36,185,438 | | 99.95% |
| Metatranscriptome | |  | |  | |  | |  | |  | |  | |  | |  | |
| CD1-midgut-1 | 50,629,489 | | 86.81% | | 6,328,686,125 | | 5,493,870,500 | | 5,274,558,564 | | 96.00% | | 9,896 | | 43,936,259 | | 86.78% |
| CD1-midgut-2 | 50,629,489 | | 86.81% | | 6,328,686,125 | | 5493870500 | | 5226991073 | | 95.14% | | 4,814 | | 43,936,259 | | 86.78% |
| CD1-hindgut-1 | 52,935,727 | | 83.59% | | 6,616,965,875 | | 5530337750 | | 5267479192 | | 95.25% | | 33,579 | | 44,201,399 | | 83.50% |
| CD1-hindgut-2 | 52,935,727 | | 83.59% | | 6,616,965,875 | | 5530337750 | | 5274953801 | | 95.38% | | 7,767 | | 44,201,399 | | 83.50% |
