# Supplementary material for: Genome Reduction in Psychromonas Species within the Gut of an Amphipod from the Ocean’s Deepest Point
Source: mSystems. 2018 Apr 10;3(3):e00009-18. doi: 10.1128/mSystems.00009-18 (PMC5893861; doi:10.1128/mSystems.00009-18)
Supplement: TABLE S2 [file sys003182223st2.docx]

**Table S2**

| Metagenome | Repeats | Spacer |
| --- | --- | --- |
| CD2 | AGAGCTCGTGTAGAGCCCGTTTAGA | GCTCGTTTAGAGTTCATGTAGAGCCCGTTG |
|  | AGAGCTCGTGTAGAGCTCGTGTAGA | GCTCGTGTAGAGCCCGTTTAGAGCTTGTGTAGAGCCCGGGT |
|  | AGAGCTCGTGTAGAGCTCGGGTAGA | GCCCGGGTAGAGCCCGTGT |
|  | AGAGCTCGTGTAGAGCTCGTGTAGA |  |
|  | Average Length: 25 | Average Length: 30 |
| CD3 | TGTACAGTGGAACGTGTGCAGT | CGAACAACAGTAGTGGAACG |
|  | TGTACAGTGGAACGTGTACAGT | CGAACAACAGCAGTGGAATGTGTACAGTGGAACG |
|  | TGTACAGTGGAACGTGTACAGT | GGAACGTGTACAGTGGAACGTGTGCAGTCGAACAACAGCAGTGGAACG |
|  | TGAGCAGTGGAACGTGTACAGT | GGAACAACCGCAGTGGAACG |
|  | TGAGCAGTGGAACGTGTGCAGT | GGAACAACAGCAGTGGAACGTGAGCAGTGGGACG |
|  | TGAGCAGTGGAACGTGTACAGT | GGAACAACCGCAGTGGAACGTGTACAGTGGAACGTGAGCAGTGGAACG |
|  | TGAGCAGTGGAACGTGAGCGGT | GGAACGTGAGCAGTGGAAAA |
|  | TGTACAGTGGAACGTGTGCAGT | CGAACAACCGCAGTGAAACG |
|  | TGAGCAGTGGAACGTGTGCAGT | GGAACGTGTGCAGTAGAACA |
|  | ACAGCAGTGGAACGTGTGTAAT |  |
|  | Average Length: 22 | Average Length: 29 |
| SD2 | CAGACAGACTGGCAGACAGACTGGTAGA | TATACAGGCAGCTAGACAGGCATACACAATGGCAGACCGACTGGCAGTCAGACTAGCAGACATACTGA |
|  | CAGACATACTGGCAGAGAGACTGGCAGA | CAGGCTAGCAGACAGACTGTTAGACAGACTGACAGACAGAATGACAGTCAGAATAG |
|  | CAGACATACTGGCAAGCAGACTGGCAGA | CATACTGGCAGACAGACTAA |
|  | CAGACAGACTGGCAGACAGACTGGCAGA | TGGACTGGTAGACAGAATAGCAGACAGACTAG |
|  | CAGACAGACTGTCAGACAGACTGGCAGA | CAGACTGGCAGGCAGACTGG |
|  | CAGAGAGACTGGCAGACAGACTGGCTGA |  |
|  | Average Length: 28 | Average Length: 39 |
| SD2 | AGACAGGCAAACAGACAGGCAAACAGACA | GGCATACAAACAGGCAAACAGACAGGGAAACAGACAGAAAAACAAACAGTCAAACAGACATACAAACAGACAAACAGACAGGCAATG |
|  | AGACAAGCAAACGGACAGGCAAGCAGACA | TGCAAACAGACAGGCAAACAGACAGGAAATAGACAGGCAAACAGAC |
|  | AGACAGGCAAACAGACAGGCAAACAGATA | GGAAAACAGACAGACATGCAAACAGACAGGAAAACAGACAGGCAAACAGACAGGCAAACAGAAAGGCAAAC |
|  | AGACAGGCAAACAGACAGGCAATCAGACA | GGCAAATAAATATGCAAACGAACAGGCAAACAGACAGACAGGCAAACAGGCAAGCAATC |
|  | AGACAGGCAAACAGAAAGGCAAACAGACA | TGCAAACAGACATGCAAAGAGACGGGCAAACAGACAGTCAAAC |
|  | AGACAGGCAAACAGACAGGCAAACAGACA | GGCAAACAGACAGGCAAACTGACAAGCAAACAGAC |
|  | AGACAGGCAAACAGACAAGCAAACAGACA | TGCAAACAGAAAGGCAAACAGACATGCAAACAGACATGCAAAGAGACAGTCAAAC |
|  | AGACAGTCAAACAGACAGGCAAACAGACA | GGCAAGCAGACATACAAACATACATGCAAAGAGATGGGCAAACAGACAGTCAAACAGACAGGCAAAC |
|  | AGACAAGCAAACAGACAGGCAAACAGACA | GGCATACAAACAGGCAAACAGACATGCAAACAAACAGGCAAACATATAGGCAAAC |
|  | AGACAAGCAAACAGACAGGCAAACAAACA | GGCAAACAGACTTGCAAACAGACAGGCAAACAGACTGGCAAAC |
|  | AGACAAGCAAACAGACAGGCAAACAGACA | AATAGACAGACAAACAGACATGCAAACAGACTGGCAAACAGAGAGGCAAAC |
|  | AAACAGGCAAACAGACGGGAAGACAGACT | GGCAAACAGACAGGCAAAC |
|  | AGACAGGCAAACAGACATGCAAACAGACA | GGCAAACAGATAGGCAAACAGACAAGCAAAC |
|  | AGACAGGCAAACGTACAGGCAAACAGACA | TGCAAACAGACAGTCAAAC |
|  | AGACAGGCAAACAGACAGGCAAACAGACA | AGCAAACAGACAGACAAACAGACAGACAAACAGACAAGCAAACAGACATGCAAAC |
|  | AGACAGGCAAACAGACAGGCAAACAGACA | GGCAAACAGACAGACAAACAGACAGACAGGCAAACAGACGGACAGGAAAC |
|  | AGACATGCAAACAGACCGGCAAACAGACT | GGCAAACAGACCTGCAAACATACAGGCCAAG |
|  | AGACAGGCAAACAGACAGGCAAACAGACA | TGCAAAGAGACAGGCAAACAGAGAGACAAACAGACCGGCAAACAGACATGCTAAC |
|  | AGACAGGCAAACAGACAGGCAAACAGACA | GGCAAA |
|  | AGACAGGCATCACCACAGCCTGAAAGACA | GGCATCACCACAACCTGAA |
|  | AGACAGGCATCACCACAGCCTGACAGACA | GGCATCATCACAGCCTGAT |
|  | AGACAGGTATCACCACGAACTGACAGACG | AGCAGCATCACAGCCTGAC |
|  | TGAAAGGTATCATCACAGCCTGACATACA | GGCATCATCACAGCCTGAA |
|  | AGACAAGCATCACCACAGCCTGACAGACA | GGCATCACCACAGCCTGATAGACAGGCATTACCACGACCTGAC |
|  | AGACATGCATTACCAAAGCCTGACAGACA | GGCATCACCACAGCCTGATGGACAGGCATCACCACGACCTGAC |
|  | AGACAGGCATCACCACAGCCTGACAGACA | GGCATCACCACGACCTGAC |
|  | AGACAGGCATCACCATAGCCTGACAGACG | ATTATCACCACGACCTGACAGACAGTCATCATCACAGCCTGAC |
|  | AGACAGGCATCACCACAGCCTGACAGACA | GGCATCACCACAGCCTGAC |
|  | AGACAGGGATCACCACGACCTGACAGACG | AGCATCACCACGACCTGACAGACAGGCATCACCACAGCCTGAC |
|  | AGACATGCATCACCACGACCTGACAGACA |  |
|  | Average Length: 29 | Average Length: 41 |
| SD2 | CCAGCCACGCACCAACCAGCCACGC | ACCAACCCGCCACGCACCAACCAGCCACGCACCAACCAGCCACGGACCAACTAGCTACACACCAA |
|  | CCAGCCACGCACCAACTAGCAACGC | ACCAACCAGCCACGCACCAACCAGCCACGCCCCAACCAGCCACGCCCCAA |
|  | CCAGCCACGCACCAACCAGCTACGT | ACCAACCCCAGCCATGCACCAACC |
|  | TTAGCCAGGCACCAACCAGCCACGC | AACAACCAGCCACTTACCAAGCAGCCACGCGCCAA |
|  | CCAGCCACGCACCAACCAGTCACGC | AACAACCAACCACGCACCAA |
|  | CCAGTCACGCACCAACCAGCCACGC | GCCAACCAGCCACGCGCCAA |
|  | CCAGCCACGCGCCAACCAACCACGC | GCCAACCGGCCACGCGCCAA |
|  | CCAACCACGCGCCAACCAACCACGC | GCCACCCAGCCACGCGCGAA |
|  | CCAGCCACGCACCAACCAGCCACGC |  |
|  | Average Length: 25 | Average Length: 31 |
| SD2 | ATTTGCACACTATTTGCACAGTATT | TGTACGGTATTTGTACACT |
|  | ATTTGCACAGTATTTGCACAGTATT | TGTACACTATTTTCACACT |
|  | ATTTGCACAGTATTTGCACAGTATT | AGCACACTATTTGTACACTATTTTCACAGT |
|  | ATTTGCACAGTATTTGCACAGTATT | TGCACACTATTTGTACACT |
|  | ATTTGCACACTATTCACACAGTATT | TGTACAGTATTTGTACACT |
|  | ATTTGCACACTATTTGCACAGTATT | TGCACAGTATTTGTACACTTTTTGAACAGTATTAGCACAGT |
|  | ATTTGTACACTATTTGTACACTATT | TGCACACTTTTTGCACAGTATTTACACACT |
|  | ATTTGCACAGTATTTGTACAGTATT | TGCACAGTATTTGCACAGTATTTGCAGAGTATTTGCACACT |
|  | ATTTGCACAGTATTTGCACAGTATT | TGCACAGTATTTGCACAGTATTTGCAGAGT |
|  | ATTTACACAGTATTTGCACAGTATT | TGAATAGTATTTGCACACTATTTGCACAGTATTTGTACACACAGT |
|  | ATTTACACAGTATTTGTACACTATT | TGTACACTAAATGCACAGTATTTGCACAGTATTTGCACAGT |
|  | ATTTGCACAGTACTTGTACACTATT | TGCACAGTATATTTCCGGTATTTTCACACT |
|  | ATTTGCACAGTATTTGGACAGTATT | TGGACAGTATTTGCACACT |
|  | ATTTGCACAGTATTTGTACACTATT | TGCATAGTATTTGCACACT |
|  | ATTTGCACAGTATTTGCACACTATT | TGGGCACTATTTGTGCACT |
|  | ATTTGCACAGTATTTGCACAATATT | TGCACAGTATTTGCACAGTAGTTGCACACTATTTGTACACT |
|  | ATTTGCACAGTATTTGCACACTATT | TGTTTACTATTTGCACAGTATTTGCACAGTATTTGAACAGTATTTGCACACT |
|  | ATTTGCACAGTATTTGCACACTATT | TGTACACTATTTTCACAGTATTTGTCCGGTATTTTCACACTATTTGCAAAGTATTTACACAGT |
|  | ATTTGCACAGTATTTACACACTATT | TGCACAGTATTTGCACACTATTTGTACACTATTCGCACAGTATTTGTCCGGT |
|  | ATTTTCACACTATTTGCACACTATT | TGCACAGTATTTGTACAGTATTTGCACAATATTTGCAAAGT |
|  | ATTTACACAGTATTTGCACAGTATT | TGCACAGTATTTGCAGATTATTAACAGAGTATTTGTACAGTATTTGTACACTATTTGCACTGTATTTGTACAGT |
|  | ATTTGCACAGTATTTGCACAGTATT | TGCACAGTATTTGTACAGTATTTGCACAGT |
|  | ATTTGCACAGTATTTGCACATTATT | TGCACAGTATCAGCACAGTATTTGCACAGTATTTGCAGAGTATTTACACAGTTTTTGTACACT |
|  | ATTTGCACAGAATTTGCACAGTATT | TGTACACTATTTTCACACT |
|  | ATTTGCACAGTATTTGCACAGTATT | TGCACACTATTTGTACACTATTTTCACAGT |
|  | ATTTGCACAGTATTTGCACAGTATT |  |
|  | Average Length: 25 | Average Length: 35 |
| SD2 | GCGTTCAGTGAGCTGATTGT | AGCGTTCAATGAACAAAGTGTAGCGTTCAGTGGGAAGAGTGTAGCGTTAAGTGTGCAGATTGTAGCGTTCAGTAAGCAGAGTGTA |
|  | GCGTTCAGTGAGCAGAGTGT | AGCGTTCAATGAGCAAAGTGTAGCGTTTAGTGAGCAGAGTGTAGCGTTCAGTGAGCAGAGTGTAGCGTACAGTGAGCTGAGTGTA |
|  | GCGTTCAGTGAGCAGAGTGT | AGCTTTCAGTGAGCAGAGTGTAGCGTTCAGTGAGCAGAGTGTAGCGTTCAGTGAGCAGAGTGTACCGTTCAGTGAGCAAAGTGTA |
|  | GCGTTCAATGAGCAGAGTGT | AGCGTTCAGTGAGCAGAGCGTAGCGTTCAATGAATAGAGTGTAGCGTTCAACGAGCAAAGTGTAGCGTTCAGTGAGTAGAGTGTA |
|  | GCGTTCAGTAAACAGAGTGT | AGCGTTCAATGAGCAGAGTGTAGCGTTCAATGAACAATGTGTAGCGTTCAGTGAGCAGAGTGTAGCGTTCAGTAACCAGAGTGTA |
|  | GCGTTCAGTAAGCAGAGTGT | ATCTTGTCTAGCAGAGTGTAGCGTTCAGTATGTAGAGTGTAGCGTTCAGTAAGCAGAGTGTATCGTGTCTGTCAGAGTGTA |
|  | GCGTTCGGTGAGCAGAGTGT | AGCGTCCAGTAAGCAGAGTGTAGCGTTCAGTGAGCAGAGTGTAGCGTTCAATGAGCAAAGTGTA |
|  | GCGTTCAGTGAGCAGAGTGT | AGCGTTCAATGAGCGAAGTTTAGCGTTCAGTGAGCAGATTGTAGCGTTCAGTAAGTAGGGTGTA |
|  | GCGTTCAGTGAGCAGAGTGT | AGCGTTCAGTGAGCAGATTGTAGCGTTTAGTGAGCAGATTGTT |
|  | GCGTTCAGTGAGCAGAGTGT | GCAGAGTGTAGCGTTCAGTGATTAGAGTGTA |
|  | GCGTTCAGTGAGCAGAGTGT | AGCGTTCAATGAGCAGAGTGTA |
|  | TCGTTCAGTGAGCAGAG>K1 |  |
|  | Average Length: 20 | Average Length: 66 |
| SD3 | GACAGCGGTAACAGTGGTGACAGT | GGTGACAGTGATAACAGTAGTAACACTGGTGACAGTGGT |
|  | GACAGTGGTGACAGTGGTGACAGT | GGTGACAGTGATGACGACAGTGGTGACAGTGGTGGCAGTGGTAGTGTGACCGTGGTGACAGTGGTACAGCGGTACAGTGGTGGC |
|  | GACAGTGATGACAGTGGTAACAGT | GGTAGTGGTAACAGTGGTGAGAGTGGTGACAGTGGTGACAGTGGT |
|  | GACAGTGGTGACAGTGGTGACACT | GGTGACACTGGTGACAGTGGTGACTGGTGACAGTGGTAACATGACAGTGGTGACACGACAGTGGTGACAC |
|  | GACAGTGGTGACGGTGGTAACAGT | GGTGACCGTGGTGACAGTGGTGACCGTGGTGACCGTGGT |
|  | GACCGTGGTGACAGTGGTGACAGT | GGCAACAGCGGTGACAGTGGTGACAAGTGGTGAAAGTGGTGACAGTGGC |
|  | GACGGTGGTGACAGTGGTAACAGT | GGTAACAGTGGTGACCGTGATGCAGGT |
|  | GACAGTGGTGACAGTGGTAACATA | TGACAAGTGGTGAAAGTGGTGACAAGTGGTAGTGGTGACAAGTTCTAGTTGT |
|  | GACGGTGGTAACAGTGGTGACAAG | TGGTGATAAGTGGTGAAGTGGTGACAAGTGGTGAAGTGGTGACAAGTGTGT |
|  | GACAGTGGTGACAGTGGTGACAGT | AAAGTAGGGACAGTGGTGACAGTAAAGTAGG |
|  | GACAGTGGTGACAGTGGTGACAGT | GGTGACAGTGTGACAGTGTT |
|  | GACAGTGGTGACAGTGGTGACAGT |  |
|  | Average Length: 24 | Average Length: 46 |
| SD3 | CAGGTTGTATTACGTGTTGT | GTTGCGTGTTGTGTTGCCCCTTGTATTGCGTGTTGTGCTGCGTGTTGGGTGTTGTGTTACGTGTGCGTGTTGCACGTTGTATTGCGTGTTGTACGTTGTATTGCGTGTTGTGTTGCGTGTGCGTGTTGCGTTTGCGTGTTG |
|  | CACGTTGTATTGCGTGTTGT | GTTGCGTGTGCGTGTTATGTTGCGTGTTGTGTTGCATGTTGTATTGCGTGTTGTGTTGCGTTCTGTGTTACACGTTGTATTGCGTGTTGTGTTGCGTGTGTGTTTATGGTGCGTTGCATGTTGTGTTGCGTGTGCGTGTTATGTTG |
|  | CGCGTTGTGTTGCGTGTTGT | GTTGCGTGTTGCGTTGCGTGTTGTGTTGCGTGTTGTGTTGCGTGTTGTGTTGCATGTTGTATTGCGTGTTGTGTTGCGCGTTGTGTTCGTGTTGTATTGCATGT |
|  | TGTGTTGTGTTGCGTGTTGT | GTTGCATGTTGTATTGCGTGTTGTGTTGCGCGGTGTGTTGCGTGTTGTATTGCGTGTTGTGTTGCGTGTGCGTGTTATGTTGCCTGTCATGTTGTGTTGCGTGTTGTGTTGCGCGTTGTGTTGTGTGTG |
|  | CGTCTTGTGTTGCGTGTTGT | GTTGCATGTTGTATTGCGTGGTGTGTTGCGTGTTGTGTTGCGTGTTGTGTTGCGCGTTGTATTGCGTGTTGTGTTGCGTGTGCGTGTTATGTTGCGTGTTGTGTTGCATGTTGTGTTG |
|  | CGTGTTGTGTTGCGCGTTGT | GTTGCGTGTGCGTCTTGTCTTGCGTGCTGTGTTGCATGTTGTATTGCATGTTGTGTTGCGTGTTGTGTTGCATCTTGTATTGCGTGTTGTGTTGCGCGGTGTGTTG |
|  | CGTGTTGTATTGCGTGTTGT | GTTGCGTGTGCGTGTTATGTTGCGTGTTGTGTTGCATGTGGTGTAGCGTGTTGTGTTGCGCGTTGTGTTGCGTGTTGTGTTGCGTGTTGTGTTGTATTG |
|  | CGTGTTGTGTTGCGTGTTGT | GTGCGTGTTGTGTTGCGCGTTGTATTGCCTGTTGTGTTGCGTGTGCGTGTTATGTTGTGTGTTGTGTTG |
|  | CATGTTGTGTTGCGTGTTGT | GTTGCGCGTTGTGTTGCGTGTTGTCTTGCGTGTGCGTATTGTGTTTGCGTGTTCTGTTGCGCGTTGTATTG |
|  | CGTGTTGTGTTGTGTGTTGT | GTTCGTGTTGTATTGCATGACGATTGTTGTGTTG |
|  | CGTATTGTGATGCGTGTTGT | GTGCGTGTTGTATGTTGTGTTGTGTTGCGTGCATGTTGTTGTTGCATGTTGTGTTG |
|  | CGTGTTGTGTTGCGTGTTGT | GTTGTGTGTTGTGTTGCGCGTTGTGTTGTGTGATGTGTTG |
|  | CGTGTTGTGTTGCGTGTTGT | GTTACGTGTTCTGTTGCGTGTTGTGTTGCGTGCATGTCGTGTTG |
|  | CATGTTGTGTTGCGTGTTGT | GTGTTGTGTTGTGTGATGTGTTG |
|  | CGTGTTGTGTTGCGTGTTGT | ATTGTGTGTTGTGTTGTGTCGCGTGTTGTGTCG |
|  | CGTGTTGTGTTGCGTGTTGT | GTGCGTGTGCGTTTTGTGTTA |
|  | CGTGTTGTGTTGCGTGTTGT | GTTGCGTGTTACGCGGTGTGCGTCGTGTCTTT |
|  | TATGTTGTGTTGTGTGGTGC |  |
|  | Average Length: 20 | Average Length: 74 |
| SD3 | CACCTGTGCGAGGCAAAACACTTG | TGCGAGGCAAAACACTCGTGAGACAAAACACCTGTGCGAGGCAAAA |
|  | CACCTGTGCGAAGCAAAACACTTG | TGCGAGGCAAAAACATTTGTGCGAAACAAAACCCTTGTGCGAGGCAAAA |
|  | CACCTGTACGACACAAAACACCTG | TGCGAGGCAAAACACCTGTGCGAGACAAAA |
|  | CACCTGTGCGAGACAAAACACCTG | CGCGATGCAAAACACCTGTGCGAGGCAAAA |
|  | CACCTGTGCGAGACAAAACACCTG | TGCGACAAAACACGCAAAA |
|  | CACCTGTGCGAGACAAAACACCTG | TGCGAGGCAAAACACCTGTGCGATGCAAAA |
|  | CACCTGTGCGATGCAAAACACCTG | TGCGATGCAAAACACCTGTGCGAGGCAAAA |
|  | CACTTGTGCGAGGCAAAACACCTG | TGCGAGACAAAACACCTGTGCTAGGTAAAACACCTGTGCGATGCAAAA |
|  | CACCTGTGCGAGGCAAAACACCTG | TGAGAGGCAAAACACCTGTGCGAGACAAAA |
|  | CACCTGTGCGAGACAAAACACCTG | TGCGAGACAAAACACATGTGCGAGACAAAACACCTGTGCGAGGCGAAA |
|  | CACCTGTGCGAGACGAAACACCTG |  |
|  | Average Length: 24 | Average Length: 36 |
