# Supplementary figures and images for: Genome Reduction in Psychromonas Species within the Gut of an Amphipod from the Ocean’s Deepest Point
Source: mSystems. 2018 Apr 10;3(3):e00009-18. doi: 10.1128/mSystems.00009-18 (PMC5893861; doi:10.1128/mSystems.00009-18)

**Figure S1**

**
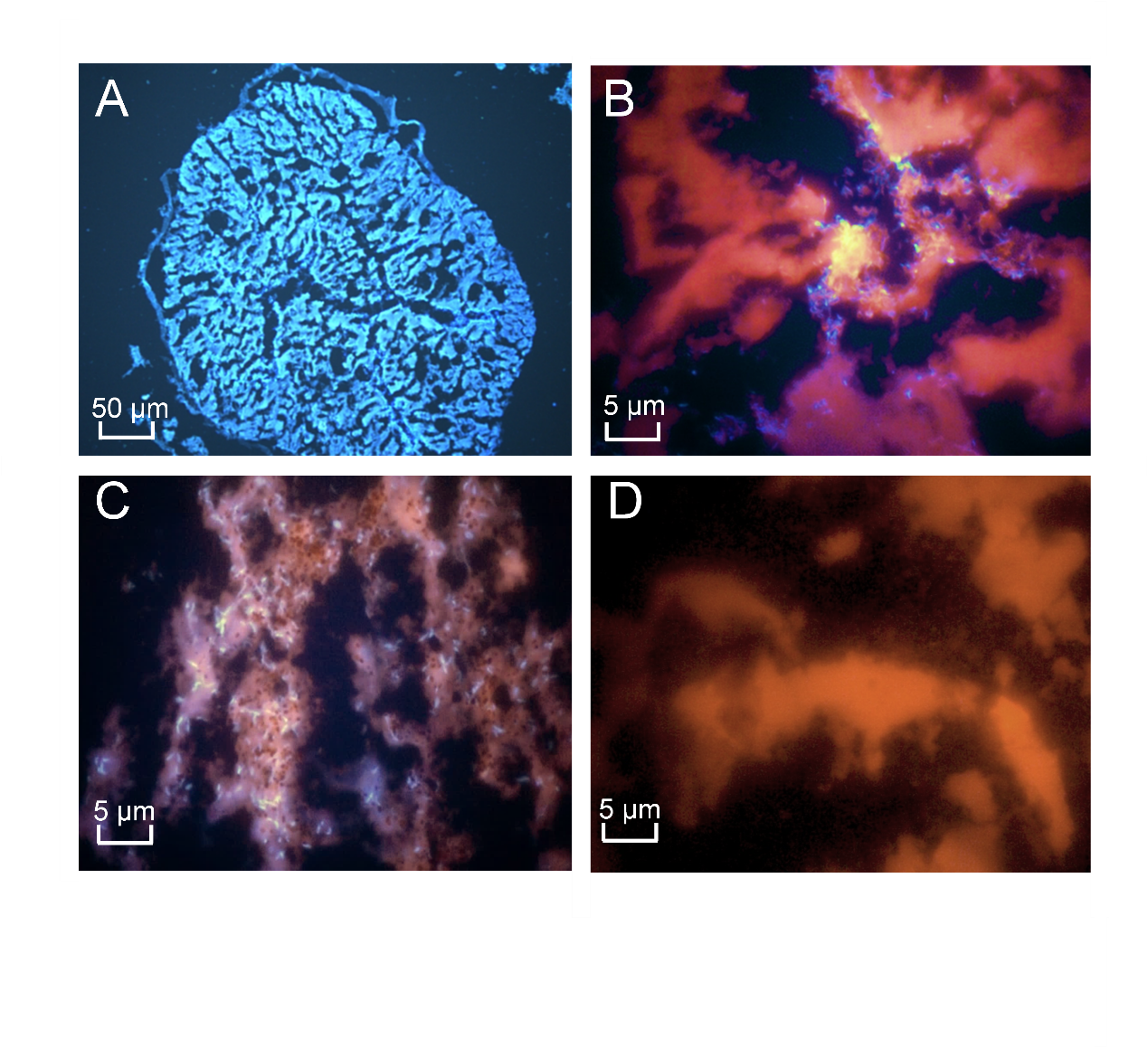
**

Supplement: FIG S1 [file sys003182223sf1.docx]
