# Supplementary material for: Genome Reduction in Psychromonas Species within the Gut of an Amphipod from the Ocean’s Deepest Point
Source: mSystems. 2018 Apr 10;3(3):e00009-18. doi: 10.1128/mSystems.00009-18 (PMC5893861; doi:10.1128/mSystems.00009-18)
Supplement: TABLE S3 [file sys003182223st3.docx]

**Table S3**

| Strains | CD2 | CD3 | CD4 | CD5 | SD1 | SD2 | SD3 | SD4 | SD5 | SD6 |
| --- | --- | --- | --- | --- | --- | --- | --- | --- | --- | --- |
| Habitat | Challenger Deep | Challenger Deep | Challenger Deep | Challenger Deep | Sirena Deep | Sirena Deep | Sirena Deep | Sirena Deep | Sirena Deep | Sirena Deep |
| Draft genome size (bp) | 34,051 | 60,893 | 73,176 | 466,865 | 1,146,436 | 49,369 | 77,082 | 47,419 | 170,706 | 156,333 |
| No. of contigs | 64 | 110 | 127 | 803 | 1,288 | 88 | 146 | 89 | 319 | 280 |
| No. of coding sequences | 78 | 140 | 172 | 997 | 2,016 | 109 | 181 | 108 | 401 | 357 |
| Intergenic length (bp) | 1,570 | 3,890 | 5,981 | 28,059 | 154,378 | 4,174 | 5,211 | 4,830 | 24,148 | 6,105 |
| GC (%) | 39.09 | 41.05 | 39.84 | 38.20 | 36.19 | 38.74 | 38.74 | 37.99 | 38.65 | 38.65 |
| ANI with CDP1 (%) | 99.83 | 97.80 | 99.20 | 99.06 | 99.71 | 99.75 | 99.33 | 99.80 | 99.73 | 99.00 |
