# Supplementary material for: Genome Reduction in Psychromonas Species within the Gut of an Amphipod from the Ocean’s Deepest Point
Source: mSystems. 2018 Apr 10;3(3):e00009-18. doi: 10.1128/mSystems.00009-18 (PMC5893861; doi:10.1128/mSystems.00009-18)
Supplement: TABLE S4 [file sys003182223st4.docx]

| **Table S4** | | | | | | |
| --- | --- | --- | --- | --- | --- | --- |
| PFAM | Annotation | *Psychromonas sp. CDP1* | *P. hadalis* | *P. ossibalaenae* | *P. aquimarina* | *P. arctica* |
| PF03772 | Competence Competence protein | 0 | 1 | 1 | 1 | 1 |
| PF00035 | DSRM dsrm Double-stranded RNA binding motif | 1 | 1 | 1 | 1 | 1 |
| PF01176 | OB eIF-1a Translation initiation factor 1A / IF-1 | 1 | 1 | 1 | 1 | 1 |
| PF06574 | HUP FAD_syn FAD synthetase | 1 | 1 | 1 | 1 | 1 |
| PF01687 | Flavokinase Riboflavin kinase | 1 | 1 | 1 | 1 | 1 |
| PF06421 | LepA_C GTP-binding protein LepA C-terminus | 1 | 1 | 1 | 1 | 1 |
| PF01252 | Peptidase_A8 Signal peptidase (SPase) II | 1 | 1 | 1 | 1 | 1 |
| PF00154 | P-loop_NTPase RecA recA bacterial DNA recombination protein | 1 | 1 | 1 | 1 | 1 |
| PF02132 | RecR RecR protein | 1 | 1 | 1 | 1 | 1 |
| PF00572 | Ribosomal_L13 Ribosomal protein L13 | 1 | 1 | 1 | 1 | 1 |
| PF01245 | KOW Ribosomal_L19 Ribosomal protein L19 | 1 | 1 | 1 | 1 | 1 |
| PF00886 | Ribosomal_S16 Ribosomal protein S16 | 1 | 1 | 1 | 1 | 1 |
| PF00380 | S5 Ribosomal_S9 Ribosomal protein S9/S16 | 1 | 1 | 1 | 1 | 1 |
| PF01782 | EFTPs RimM RimM N-terminal domain | 1 | 1 | 1 | 1 | 1 |
| PF02403 | tRNA_bind_arm Seryl_tRNA_N Seryl-tRNA synthetase N-terminal domain | 1 | 1 | 1 | 1 | 1 |
| PF00763 | AA_dh_N THF_DHG_CYH Tetrahydrofolate dehydrogenase/cyclohydrolase, catalytic domain | 1 | 1 | 1 | 1 | 1 |
| PF02882 | NADP_Rossmann THF_DHG_CYH_C Tetrahydrofolate dehydrogenase/cyclohydrolase, NAD(P)-binding domain | 1 | 1 | 1 | 1 | 1 |
| PF03461 | TRCF TRCF domain | 1 | 1 | 1 | 1 | 1 |
| PF01746 | SPOUT tRNA_m1G_MT tRNA (Guanine-1)-methyltransferase | 1 | 1 | 1 | 1 | 1 |
| PF02367 | P-loop_NTPase TsaE Threonylcarbamoyl adenosine biosynthesis protein TsaE | 1 | 0 | 0 | 0 | 0 |
| PF08459 | Endonuclease UvrC_HhH_N UvrC Helix-hairpin-helix N-terminal | 1 | 1 | 1 | 1 | 1 |
| PF03485 | Arg_tRNA_synt_N Arginyl tRNA synthetase N terminal domain | 1 | 1 | 1 | 1 | 1 |
| PF03484 | B5 tRNA synthetase B5 domain | 1 | 1 | 1 | 1 | 1 |
| PF01121 | P-loop_NTPase CoaE Dephospho-CoA kinase | 1 | 1 | 1 | 1 | 1 |
| PF03602 | NADP_Rossmann Cons_hypoth95 Conserved hypothetical protein 95 | 1 | 1 | 1 | 1 | 1 |
| PF02224 | P-loop_NTPase Cytidylate_kin Cytidylate kinase | 1 | 1 | 1 | 1 | 1 |
| PF00712 | DNA_clamp DNA_pol3_beta DNA polymerase III beta subunit, N-terminal domain | 1 | 1 | 1 | 1 | 1 |
| PF02767 | DNA_clamp DNA_pol3_beta_2 DNA polymerase III beta subunit, central domain | 1 | 1 | 1 | 1 | 1 |
| PF02768 | DNA_clamp DNA_pol3_beta_3 DNA polymerase III beta subunit, C-terminal domain | 1 | 1 | 1 | 1 | 1 |
| PF00889 | EF_TS Elongation factor TS | 1 | 1 | 1 | 1 | 1 |
| PF03147 | FDX-ACB Ferredoxin-fold anticodon binding domain | 1 | 1 | 1 | 1 | 1 |
| PF02938 | GAD GAD GAD domain | 1 | 1 | 1 | 1 | 1 |
| PF02527 | NADP_Rossmann GidB rRNA small subunit methyltransferase G | 1 | 1 | 1 | 1 | 1 |
| PF00958 | GMP_synt_C GMP synthase C terminal domain | 1 | 1 | 1 | 1 | 1 |
| PF01025 | GrpE GrpE | 1 | 1 | 1 | 1 | 1 |
| PF01018 | GTP1_OBG GTP1/OBG | 1 | 1 | 1 | 1 | 1 |
| PF11987 | IF-2 Translation-initiation factor 2 | 1 | 1 | 1 | 1 | 1 |
| PF00707 | IF3_C Translation initiation factor IF-3, C-terminal domain | 1 | 1 | 1 | 1 | 1 |
| PF05198 | IF3_N Translation initiation factor IF-3, N-terminal domain | 1 | 1 | 1 | 1 | 1 |
| PF01715 | P-loop_NTPase IPPT IPP transferase | 1 | 1 | 1 | 1 | 1 |
| PF01795 | NADP_Rossmann Methyltransf_5 MraW methylase family | 1 | 1 | 1 | 1 | 1 |
| PF02873 | MurB_C UDP-N-acetylenolpyruvoylglucosamine reductase, C-terminal domain | 1 | 1 | 1 | 1 | 1 |
| PF08529 | NusA_N NusA N-terminal domain | 1 | 1 | 1 | 1 | 1 |
| PF02410 | NTP_transf RsfS Ribosomal silencing factor during starvation | 1 | 1 | 1 | 1 | 1 |
| PF00162 | PGK Phosphoglycerate kinase | 1 | 1 | 1 | 1 | 1 |
| PF02912 | tRNA_bind_arm Phe_tRNA-synt_N Aminoacyl tRNA synthetase class II, N-terminal domain | 1 | 1 | 1 | 1 | 1 |
| PF03726 | PNPase Polyribonucleotide nucleotidyltransferase, RNA binding domain | 1 | 1 | 1 | 1 | 1 |
| PF02033 | RBFA Ribosome-binding factor A | 1 | 1 | 1 | 1 | 1 |
| PF00687 | Ribosomal_L1 Ribosomal protein L1p/L10e family | 1 | 1 | 1 | 1 | 1 |
| PF00466 | Ribosomal_L10 Ribosomal protein L10 | 1 | 1 | 1 | 1 | 1 |
| PF00298 | Ribosomal_L11 Ribosomal protein L11, RNA binding domain | 1 | 1 | 1 | 1 | 1 |
| PF03946 | Ribosomal_L11_N Ribosomal protein L11, N-terminal domain | 1 | 1 | 1 | 1 | 1 |
| PF00542 | Ribosomal_L12 Ribosomal protein L7/L12 C-terminal domain | 1 | 1 | 1 | 1 | 1 |
| PF00252 | Ribosomal_L16 Ribosomal protein L16p/L10e | 1 | 1 | 1 | 1 | 1 |
| PF01196 | Ribosomal_L17 Ribosomal protein L17 | 1 | 1 | 1 | 1 | 1 |
| PF00828 | Ribos_L15p_L18e Ribosomal_L27A Ribosomal proteins 50S-L15, 50S-L18e, 60S-L27A | 1 | 1 | 1 | 1 | 1 |
| PF00181 | OB Ribosomal_L2 Ribosomal Proteins L2, RNA binding domain | 1 | 1 | 1 | 1 | 1 |
| PF03947 | KOW Ribosomal_L2_C Ribosomal Proteins L2, C-terminal domain | 1 | 1 | 1 | 1 | 1 |
| PF00453 | Ribosomal_L20 Ribosomal protein L20 | 1 | 1 | 1 | 1 | 1 |
| PF00829 | Ribosomal_L21p Ribosomal prokaryotic L21 protein | 1 | 1 | 1 | 1 | 1 |
| PF00237 | Ribosomal_L22 Ribosomal protein L22p/L17e | 1 | 1 | 1 | 1 | 1 |
| PF00276 | Ribosomal_L23 Ribosomal protein L23 | 1 | 1 | 1 | 1 | 1 |
| PF01016 | Ribosomal_L27 Ribosomal L27 protein | 1 | 1 | 1 | 1 | 1 |
| PF00830 | Ribosomal_L28 Ribosomal L28 family | 1 | 1 | 1 | 1 | 1 |
| PF00831 | Ribo_L29 Ribosomal_L29 Ribosomal L29 protein | 1 | 1 | 1 | 1 | 1 |
| PF00297 | EFTPs Ribosomal_L3 Ribosomal protein L3 | 1 | 1 | 1 | 1 | 1 |
| PF01783 | Zn_Beta_Ribbon Ribosomal_L32p Ribosomal L32p protein family | 1 | 1 | 1 | 1 | 1 |
| PF01632 | Ribosomal_L35p Ribosomal protein L35 | 1 | 1 | 1 | 1 | 1 |
| PF00573 | Ribosomal_L4 Ribosomal protein L4/L1 family | 1 | 1 | 1 | 1 | 1 |
| PF03948 | Ribosomal_L9_C Ribosomal protein L9, C-terminal domain | 1 | 1 | 1 | 1 | 1 |
| PF01281 | Ribosomal_L9_N Ribosomal protein L9, N-terminal domain | 1 | 1 | 1 | 1 | 1 |
| PF00338 | Ribosomal_S10 Ribosomal protein S10p/S20e | 1 | 1 | 1 | 1 | 1 |
| PF00411 | S11_L18p Ribosomal_S11 Ribosomal protein S11 | 1 | 1 | 1 | 1 | 1 |
| PF00164 | OB Ribosom_S12_S23 Ribosomal protein S12/S23 | 1 | 1 | 1 | 1 | 1 |
| PF00416 | H2TH Ribosomal_S13 Ribosomal protein S13/S18 | 1 | 1 | 1 | 1 | 1 |
| PF00312 | S15_NS1 Ribosomal_S15 Ribosomal protein S15 | 1 | 1 | 1 | 1 | 1 |
| PF00366 | OB Ribosomal_S17 Ribosomal protein S17 | 1 | 1 | 1 | 1 | 1 |
| PF01084 | Ribosomal_S18 Ribosomal protein S18 | 1 | 1 | 1 | 1 | 1 |
| PF00203 | Ribosomal_S19 Ribosomal protein S19 | 1 | 1 | 1 | 1 | 1 |
| PF00318 | Ribosomal_S2 Ribosomal protein S2 | 1 | 1 | 1 | 1 | 1 |
| PF01649 | Ribosomal_S20p Ribosomal protein S20 | 1 | 1 | 1 | 1 | 1 |
| PF00189 | Ribosomal_S3_C Ribosomal protein S3, C-terminal domain | 1 | 1 | 1 | 1 | 1 |
| PF00163 | S4 Ribosomal_S4 Ribosomal protein S4/S9 N-terminal domain | 1 | 1 | 1 | 1 | 1 |
| PF01250 | Ribosomal_S6 Ribosomal protein S6 | 1 | 1 | 1 | 1 | 1 |
| PF01000 | RNA_pol_A_bac RNA polymerase Rpb3/RpoA insert domain | 1 | 1 | 1 | 1 | 1 |
| PF03118 | HHH RNA_pol_A_CTD Bacterial RNA polymerase, alpha chain C terminal domain | 1 | 1 | 1 | 1 | 1 |
| PF01193 | RBP11-like RNA_pol_L RNA polymerase Rpb3/Rpb11 dimerisation domain | 1 | 1 | 1 | 1 | 1 |
| PF04997 | RNA_pol_Rpb1_1 RNA polymerase Rpb1, domain 1 | 1 | 1 | 1 | 1 | 1 |
| PF00623 | RNA_pol_Rpb1_2 RNA polymerase Rpb1, domain 2 | 1 | 1 | 1 | 1 | 1 |
| PF04983 | RNA_pol_Rpb1_3 RNA polymerase Rpb1, domain 3 | 1 | 1 | 1 | 1 | 1 |
| PF05000 | RNA_pol_Rpb1_4 RNA polymerase Rpb1, domain 4 | 1 | 1 | 1 | 1 | 1 |
| PF04998 | RNA_pol_Rpb1_5 RNA polymerase Rpb1, domain 5 | 1 | 1 | 1 | 1 | 1 |
| PF04563 | RNA_pol_Rpb2_1 RNA polymerase beta subunit | 1 | 1 | 1 | 1 | 1 |
| PF04565 | RNA_pol_Rpb2_3 RNA polymerase Rpb2, domain 3 | 1 | 1 | 1 | 1 | 1 |
| PF10385 | RNA_pol_Rpb2_45 RNA polymerase beta subunit external 1 domain | 1 | 1 | 1 | 1 | 1 |
| PF00562 | LEF-8-like RNA_pol_Rpb2_6 RNA polymerase Rpb2, domain 6 | 1 | 1 | 1 | 1 | 1 |
| PF04560 | RNA_pol_Rpb2_7 RNA polymerase Rpb2, domain 7 | 1 | 1 | 1 | 1 | 1 |
| PF01765 | RRF Ribosome recycling factor | 1 | 1 | 1 | 1 | 1 |
| PF02773 | S-AdoMet_synt_C S-adenosylmethionine synthetase, C-terminal domain | 1 | 1 | 1 | 1 | 1 |
| PF02772 | S-AdoMet_synt_M S-adenosylmethionine synthetase, central domain | 1 | 1 | 1 | 1 | 1 |
| PF00584 | SecE SecE/Sec61-gamma subunits of protein translocation complex | 1 | 1 | 1 | 1 | 1 |
| PF03840 | SecG Preprotein translocase SecG subunit | 1 | 1 | 1 | 1 | 1 |
| PF00344 | SecY SecY translocase | 1 | 1 | 1 | 1 | 1 |
| PF01668 | SmpB SmpB protein | 1 | 1 | 1 | 1 | 1 |
| PF02978 | SRP_SPB Signal peptide binding domain | 1 | 1 | 1 | 1 | 1 |
| PF00121 | TIM_barrel TIM Triosephosphate isomerase | 1 | 1 | 1 | 1 | 1 |
| PF08275 | Toprim_N DNA primase catalytic core, N-terminal domain | 1 | 1 | 1 | 1 | 1 |
| PF05698 | Trigger_C Trigger_C Bacterial trigger factor protein (TF) C-terminus | 1 | 1 | 1 | 1 | 1 |
| PF05697 | Trigger_N Bacterial trigger factor protein (TF) | 1 | 1 | 1 | 1 | 1 |
| PF00750 | HUP tRNA-synt_1d tRNA synthetases class I (R) | 1 | 1 | 1 | 1 | 1 |
| PF01409 | tRNA_synt_II tRNA-synt_2d tRNA synthetases class II core domain (F) | 1 | 1 | 1 | 1 | 1 |
| PF01509 | TruB_N TruB family pseudouridylate synthase (N terminal domain) | 1 | 1 | 1 | 1 | 1 |
| PF00627 | UBA UBA UBA/TS-N domain | 1 | 1 | 1 | 1 | 1 |
| PF02130 | UPF0054 Uncharacterized protein family UPF0054 | 1 | 0 | 0 | 0 | 0 |
| PF03652 | YqgF RuvX Holliday junction resolvase | 1 | 0 | 0 | 0 | 0 |
| PF12344 | UvrB Ultra-violet resistance protein B | 1 | 1 | 1 | 1 | 1 |
| PF10458 | tRNA_bind_arm Val_tRNA-synt_C Valyl tRNA synthetase tRNA binding arm | 1 | 1 | 1 | 1 | 1 |
| PF06071 | Ubiquitin YchF-GTPase_C Protein of unknown function (DUF933) | 1 | 1 | 1 | 1 | 1 |
| PF06689 | zf-C4_ClpX ClpX C4-type zinc finger | 1 | 1 | 1 | 1 | 1 |
| PF06418 | P-loop_NTPase CTP_synth_N CTP synthase N-terminus | 1 | 1 | 1 | 1 | 1 |
| PF00113 | Enolase_TIM Enolase_C Enolase, C-terminal TIM barrel domain | 1 | 1 | 1 | 1 | 1 |
| PF03952 | Enolase_N Enolase_N Enolase, N-terminal domain | 1 | 1 | 1 | 1 | 1 |
| PF04760 | HTH IF2_N Translation initiation factor IF-2, N-terminal region | 2 | 1 | 1 | 1 | 1 |
| PF01195 | Pept_tRNA_hydro Peptidyl-tRNA hydrolase | 1 | 1 | 1 | 1 | 1 |
| PF01416 | PseudoU_synth_1 tRNA pseudouridine synthase | 2 | 1 | 1 | 1 | 1 |
| PF00825 | S5 Ribonuclease_P Ribonuclease P | 1 | 1 | 1 | 1 | 1 |
| PF00238 | Ribosomal_L14 Ribosomal protein L14p/L23e | 1 | 1 | 1 | 1 | 1 |
| PF00861 | S11_L18p Ribosomal_L18p Ribosomal L18 of archaea, bacteria, mitoch. and chloroplast | 1 | 1 | 1 | 1 | 1 |
| PF00281 | Ribosomal_L5 Ribosomal protein L5 | 1 | 1 | 1 | 1 | 1 |
| PF00673 | Ribosomal_L5_C ribosomal L5P family C-terminus | 1 | 1 | 1 | 1 | 1 |
| PF00333 | DSRM Ribosomal_S5 Ribosomal protein S5, N-terminal domain | 1 | 1 | 1 | 1 | 1 |
| PF03719 | S5 Ribosomal_S5_C Ribosomal protein S5, C-terminal domain | 1 | 1 | 1 | 1 | 1 |
| PF00177 | Ribosomal_S7 Ribosomal protein S7p/S5e | 1 | 1 | 1 | 1 | 1 |
| PF00410 | Ribosomal_S8 Ribosomal protein S8 | 1 | 1 | 1 | 1 | 1 |
| PF04561 | RNA_pol_Rpb2_2 RNA polymerase Rpb2, domain 2 | 2 | 1 | 1 | 1 | 1 |
| PF07499 | UBA RuvA_C RuvA, C-terminal domain | 1 | 1 | 1 | 1 | 1 |
| PF01330 | OB RuvA_N RuvA N terminal domain | 1 | 1 | 1 | 1 | 1 |
| PF05491 | HTH RuvB_C Holliday junction DNA helicase ruvB C-terminus | 1 | 1 | 1 | 1 | 1 |
| PF00347 | Ribosomal_L6 Ribosomal protein L6 | 2 | 1 | 1 | 1 | 1 |
