# Supplementary material for: Genome Reduction in Psychromonas Species within the Gut of an Amphipod from the Ocean’s Deepest Point
Source: mSystems. 2018 Apr 10;3(3):e00009-18. doi: 10.1128/mSystems.00009-18 (PMC5893861; doi:10.1128/mSystems.00009-18)
Supplement: TABLE S5 [file sys003182223st5.docx]

**Table S5**

| KEGG | Function |
| --- | --- |
| K12138 | *hyfC*; hydrogenase-4 component C [EC:1.-.-.-] |
| K02598 | *nirC*; nitrite transporter |
| K01312 | PRSS1_2_3; trypsin [EC:3.4.21.4] |
| K03833 | *selB*, EEFSEC; selenocysteine-specific elongation factor |
| K06141 | *tsgA*; MFS transporter, TsgA protein |
| K01478 | *arcA*; arginine deiminase [EC:3.5.3.6] |
| K08659 | *pepDA*, *pepDB*; dipeptidase [EC:3.4.-.-] |
| K10123 | *feoC*; putative ferrous iron transport protein C |
| K00782 | *lldG*; L-lactate dehydrogenase complex protein LldG |
| K12137 | *hyfB*; hydrogenase-4 component B [EC:1.-.-.-] |
| K03620 | *hyaC*; Ni/Fe-hydrogenase 1 B-type cytochrome subunit |
| K07812 | *torZ*; trimethylamine-N-oxide reductase (cytochrome c) [EC:1.7.2.3] |
| K08302 | *gatY*-*kbaY*; tagatose 1,6-diphosphate aldolase GatY/KbaY [EC:4.1.2.40] |
| K00613 | GATM; glycine amidinotransferase [EC:2.1.4.1] |
| K12146 | *hyfR*; hydrogenase-4 transcriptional activator |
| K05927 | *hydA*; quinone-reactive Ni/Fe-hydrogenase small subunit [EC:1.12.5.1] |
| K03192 | *ureJ*; urease accessory protein |
| K08315 | *hycI*; hydrogenase 3 maturation protease [EC:3.4.23.51] |
| K12141 | *hyfF*; hydrogenase-4 component F [EC:1.-.-.-] |
| K12136 | *hyfA*; hydrogenase-4 component A [EC:1.-.-.-] |
| K02779 | PTS-Glc-EIIC, ptsG; PTS system, glucose-specific IIC component |
| K12140 | *hyfE*; hydrogenase-4 component E [EC:1.-.-.-] |
| K14407 | CSTF2, RNA15; cleavage stimulation factor subunit 2 |
| K01152 | isftu1; isfu1 transposase |
| K00972 | UAP1; UDP-N-acetylglucosamine/UDP-N-acetylgalactosamine diphosphorylase |
| K06909 | *xtmB*; phage terminase large subunit |
| K05796 | *hydN*; electron transport protein HydN |
| K12145 | *hyfJ*; hydrogenase-4 component J [EC:1.-.-.-] |
| K05922 | *hydB*; quinone-reactive Ni/Fe-hydrogenase large subunit [EC:1.12.5.1] |
| K01274 | E3.4.13.-; D-alanyl-D-alanine dipeptidase [EC:3.4.13.-] |
| K03549 | *kup*; KUP system potassium uptake protein |
| K01618 | K01618; 3,4-dihydroxyphenylacetaldehyde synthase [EC:4.1.1.-] |
| K01042 | *selA*; L-seryl-tRNA(Ser) seleniumtransferase [EC:2.9.1.1] |
| K06666 | TUP1; general transcriptional corepressor TUP1 |
| K11436 | PRMT3; type I protein arginine methyltransferase [EC:2.1.1.319] |
| K12139 | *hyfD*; hydrogenase-4 component D [EC:1.-.-.-] |
| K03605 | *hyaD*, hybD; hydrogenase maturation protease [EC:3.4.23.-] |
| K01755 | *argH*, ASL; argininosuccinate lyase [EC:4.3.2.1] |
| K12143 | *hyfH*; hydrogenase-4 component H |
| K01551 | *arsA*, ASNA1; arsenite-transporting ATPase [EC:3.6.3.16] |
