# Supplementary material for: Genome Reduction in Psychromonas Species within the Gut of an Amphipod from the Ocean’s Deepest Point
Source: mSystems. 2018 Apr 10;3(3):e00009-18. doi: 10.1128/mSystems.00009-18 (PMC5893861; doi:10.1128/mSystems.00009-18)
Supplement: TABLE S6 [file sys003182223st6.docx]

| **Table S6** | | | | | |
| --- | --- | --- | --- | --- | --- |
| Carbohydrate metabolism pathways | *Psychromonas* sp. CDP1 | *P. hadalis* | *P. ossibalaenae* | *P. aquimarina* | *P. arctica* |
| Acetoin, butanediol metabolism | 0 | 4 | 5 | 5 | 5 |
| Acetone butanol ethanol synthesis | 2 | 5 | 5 | 3 | 4 |
| Acetyl-CoA fermentation to butyrate | 1 | 1 | 2 | 2 | 2 |
| Acinetobacter TCA | 11 | 17 | 15 | 16 | 21 |
| Alpha carboxysome | 2 | 3 | 4 | 4 | 5 |
| Alpha-amylase locus in *Streptocococcus* | 0 | 3 | 4 | 5 | 1 |
| Beta carboxysome | 0 | 2 | 7 | 9 | 3 |
| Beta-glucoside metabolism | 0 | 0 | 23 | 15 | 5 |
| Butanol biosynthesis | 2 | 3 | 4 | 5 | 5 |
| Calvin-Benson cycle | 5 | 15 | 14 | 15 | 13 |
| Carboxysome | 1 | 4 | 9 | 12 | 3 |
| Chitin and N-acetylglucosamine utilization | 5 | 22 | 44 | 29 | 9 |
| Citrate metabolism KE | 3 | 5 | 12 | 13 | 10 |
| Citrate metabolism KE3 | 5 | 6 | 6 | 6 | 9 |
| Citrate metabolism KE4 | 5 | 6 | 6 | 6 | 11 |
| CO2 uptake, carboxysome | 0 | 2 | 7 | 9 | 3 |
| D-allose utilization | 0 | 0 | 0 | 0 | 3 |
| Dehydrogenase complexes | 4 | 4 | 4 | 7 | 4 |
| Deoxyribose and deoxynucleoside catabolism | 5 | 10 | 13 | 12 | 9 |
| D-galactarate, D-glucarate and D-glycerate catabolism | 1 | 5 | 3 | 3 | 5 |
| D-galacturonate and D-glucuronate utilization | 6 | 20 | 19 | 19 | 14 |
| D-gluconate and ketogluconates metabolism | 1 | 11 | 6 | 5 | 12 |
| Dihydroxyacetone kinases | 0 | 4 | 1 | 1 | 1 |
| D-mannitol and D-mannose degradation in plants | 0 | 1 | 5 | 4 | 1 |
| D-ribose utilization | 2 | 12 | 8 | 7 | 8 |
| D-sorbitol(D-glucitol) and L-sorbose utilization | 0 | 1 | 1 | 1 | 0 |
| Entner-Doudoroff pathway | 6 | 26 | 23 | 21 | 27 |
| Ethanolamine utilization | 2 | 9 | 3 | 3 | 12 |
| Ethylmalonyl-CoA pathway of C2 assimilation | 0 | 0 | 0 | 0 | 1 |
| Fermentations in *Streptococci* | 10 | 22 | 18 | 18 | 26 |
| Fermentations: lactate | 5 | 12 | 11 | 9 | 15 |
| Fermentations: mixed acid | 8 | 18 | 16 | 15 | 18 |
| Formaldehyde assimilation: ribulose monophosphate pathway | 4 | 11 | 8 | 8 | 11 |
| Fructooligosaccharides(FOS) and raffinose utilization | 0 | 0 | 15 | 12 | 6 |
| Fructose utilization | 4 | 11 | 23 | 21 | 12 |
| Galactose degradation in plants | 4 | 11 | 12 | 11 | 7 |
| Glycerate metabolism | 1 | 6 | 4 | 4 | 8 |
| Glycerol and glycerol-3-phosphate uptake and utilization | 2 | 3 | 14 | 14 | 15 |
| Glycerol fermentation to 1,3-propanediol | 0 | 6 | 3 | 3 | 8 |
| Glycolate, glyoxylate interconversions | 0 | 3 | 1 | 1 | 3 |
| Glycolysis and gluconeogenesis | 8 | 19 | 16 | 17 | 20 |
| Glyoxylate bypass | 2 | 3 | 4 | 4 | 6 |
| Hexose phosphate uptake system | 0 | 0 | 9 | 5 | 3 |
| Inositol catabolism | 0 | 1 | 0 | 0 | 1 |
| Isobutyryl-CoA to propionyl-CoA module | 0 | 0 | 0 | 3 | 1 |
| Lactate utilization | 0 | 0 | 1 | 0 | 5 |
| Lactate utilization temp | 1 | 2 | 2 | 1 | 9 |
| Lacto-n-biose I and galacto-N-biose metabolicpathway | 2 | 5 | 5 | 6 | 3 |
| Lactose and galactose uptake and utilization | 4 | 18 | 14 | 13 | 15 |
| Lactose utilization | 0 | 8 | 5 | 3 | 3 |
| L-arabinose utilization | 0 | 0 | 1 | 1 | 2 |
| L-ascorbate utilization (and related gene clusters) | 0 | 1 | 1 | 1 | 1 |
| L-fucose utilization | 0 | 9 | 0 | 0 | 0 |
| L-rhamnose utilization | 1 | 5 | 2 | 1 | 2 |
| Maltose and maltodextrin utilization | 0 | 23 | 40 | 36 | 26 |
| Mannitol utilization | 0 | 4 | 7 | 6 | 7 |
| Mannose metabolism | 1 | 7 | 23 | 18 | 4 |
| Melibiose utilization | 0 | 0 | 7 | 5 | 1 |
| Methylcitrate cycle | 1 | 2 | 2 | 4 | 8 |
| Methylglyoxal metabolism | 1 | 5 | 5 | 5 | 8 |
| N-acetyl-galactosamine and galactosamine utilization | 3 | 18 | 22 | 16 | 1 |
| One-carbon metabolism by tetrahydropterines | 1 | 4 | 5 | 6 | 4 |
| Pentose phosphate pathway | 3 | 8 | 15 | 15 | 12 |
| Peripheral glucose catabolism pathways | 0 | 2 | 1 | 1 | 4 |
| Photorespiration (oxidative C2 cycle) | 3 | 12 | 7 | 8 | 17 |
| Propanediol utilization | 0 | 4 | 1 | 1 | 4 |
| Propionate-CoA to succinate module | 1 | 1 | 1 | 2 | 8 |
| Pyruvate alanine serine interconversions | 2 | 6 | 9 | 10 | 11 |
| Pyruvate metabolism I: anaplerotic reactions, PEP | 3 | 11 | 11 | 11 | 11 |
| Pyruvate metabolism II: acetyl-CoA, acetogenesis from pyruvate | 6 | 15 | 14 | 17 | 15 |
| Pyruvate:ferredoxin oxidoreductase | 0 | 0 | 1 | 1 | 0 |
| Quinones HGM | 6 | 7 | 8 | 8 | 9 |
| Racemases and epimerases acting on carbohydrates and derivatives | 2 | 8 | 9 | 9 | 5 |
| Serine-glyoxylate cycle | 11 | 16 | 19 | 20 | 24 |
| Sucrose utilization | 0 | 0 | 6 | 5 | 7 |
| Sucrose utilization in *Shewanella* | 0 | 0 | 1 | 1 | 2 |
| TCA cycle | 11 | 17 | 15 | 16 | 21 |
| Trehalose biosynthesis | 0 | 5 | 5 | 7 | 4 |
| Trehalose uptake and utilization | 1 | 9 | 17 | 15 | 8 |
| Unknown carbohydrate utilization (cluster Ydj) | 0 | 0 | 0 | 0 | 1 |
| Unknown sugar utilization (cluster *yphABCDEFG*) | 0 | 0 | 1 | 0 | 0 |
| Xylose utilization | 0 | 0 | 5 | 1 | 2 |
| (GlcNAc)2 catabolic operon | 0 | 8 | 9 | 9 | 0 |
| 2-ketogluconate utilization | 0 | 0 | 1 | 1 | 0 |
| 2-O-alpha-mannosyl-D-glycerate utilization | 0 | 0 | 3 | 3 | 2 |
| Total | 185 | 577 | 721 | 678 | 642 |
